# One anastomosis gastric bypass (OAGB): a scoping review

**DOI:** 10.1186/s12893-025-03215-x

**Published:** 2025-10-10

**Authors:** Jesús Elías Ortíz-Gómez, Alberto Iván González-Barajas, Jorge Alberto Guevara-Díaz, Javier Mancilla-Galindo, Ashuin Kammar-García, Manuel Alberto Guerrero-Gutierrez

**Affiliations:** 1 Department of Bariatric Surgery, Bariatric Medical Center, Tijuana, Baja California Mexico; 2 Medical Resident, Hospital General Regional #1, Instituto Mexicano del Servicio Social, Culiacan, Sinaloa Mexico; 3 Institute for Risk Assessment Sciences, Utrecht University, Utrecht, Netherlands; 4 Dirección de Investigación, Instituto Nacional de Geriatría, Mexico City, Mexico; 5 Department of Global Health and Population, Harvard TH Chan School of Public Health, Boston, United States of America; 6 Department of Anesthesiology and Intensive Care, Bariatric Medical Center, Tijuana, Baja California Mexico

**Keywords:** Bariatric surgery, Obesity management, Gastric bypass, OAGB, Mini gastric bypass, Diabetes remission, Postoperative complications

## Abstract

**Background:**

Obesity has become a global health crisis, with bariatric surgery being among the most effective interventions for treatment-resistant obesity. One Anastomosis Gastric Bypass (OAGB) has gained popularity for its technical simplicity and promising outcomes. However, concerns remain regarding long-term complications, especially bile reflux and gastroesophageal reflux disease (GERD). This scoping review aimed to synthesize the current literature on OAGB, focusing on four domains: percentage excess weight loss (%EWL), remission of metabolic and cardiovascular conditions, postoperative complications, and incidence of GERD.

**Methods:**

A systematic search was conducted in PubMed, Embase, and Web of Science (May 2025). We included randomized and non-randomized trials, systematic reviews, meta-analyses, and observational studies that reported at least one of the target outcomes in adults undergoing OAGB. Case reports, narrative reviews, case series, and retracted studies were excluded. Screening was performed using the Active Learning for Systematic Reviews (ASReview) tool. Data were charted narratively and summarized in tables. Definitions of outcomes and surgical variations were recorded where available. Risk of bias in novel randomized controlled trials was assessed using Cochrane’s RoB 2.0 tool.

**Results:**

Sixty-seven studies were included out of 3791 records screened. OAGB showed higher or comparable %EWL versus Roux-en-Y gastric bypass (RYGB), sleeve gastrectomy (SG), and SADI-S, particularly in short- and mid-term follow-up. However, there was significant variability in how %EWL was defined and calculated, including inconsistent or absent definitions of ideal weight. Type 2 diabetes remission ranged from 76.8 to 100%, with meaningful improvements in hypertension, dyslipidemia, and obstructive sleep apnea. GERD incidence varied and was influenced by limb length, presence of hiatal hernia, and surgical technique. Longer biliopancreatic limbs enhanced weight loss but increased nutritional risk. Definitions and reporting of complications varied significantly.

**Conclusions:**

OAGB is an effective metabolic and bariatric procedure with favorable outcomes in weight loss and disease remission. However, heterogeneity in surgical techniques, outome definitions, and limited follow-up time to assess long-term outcomes emphasize the need for standardized reporting and further high-quality long-term studies to guide patient selection and decision making.

**Supplementary Information:**

The online version contains supplementary material available at 10.1186/s12893-025-03215-x.

## Introduction

Obesity has become a global health crisis, with its prevalence increasing in recent decades [1]. Compared to non-operative management, metabolic and bariatric surgery (MBS) has emerged as the most effective therapeutic approach for refractory clinical obesity and associated medical problems [2]. Roux-en-Y gastric bypass (RYGB) has long been one of the most established and widely studied procedures in MBS [3]. However, the one-anastomosis gastric bypass (OAGB), also known as mini-gastric bypass (MGB), has rapidly gained traction since its introduction due to its technical advantages and long-term effectiveness.

The OABG technique has historical roots dating back to 1967, when Mason et al. [4] performed a loop bypass based on Billroth II surgery, which was later considered too radical. In 1997, Dr. Rudledge introduced what was initially termed the MGB, simplifying the procedure by creating a narrow, long gastric reservoir with a single anastomosis to the jejunum, reducing surgical time and complications [5]. Although this procedure, now formally referred to as OAGB, demonstrated advantages such as lower risk of hernias and intestinal obstruction, concerns about bile reflux remained over time [6, 7]. In 2002, Dr. Miguel Carbajo introduced a modification aimed at reducing bile reflux by laterally suturing the afferent jejunal limb. Although the technique gained acceptance, evidence supporting a definitive reduction in GERD remains limited [6, 8]. 

Compared to standard RYGB, OAGB is technically simpler, requires a shorter operative time, and has been associated with comparable or superior weight loss and type 2 diabetes remission [9]. However, an increased risk of nutritional complications and bile reflux has also been found [10], which has generated concern and controversy about its general safety, thus limiting its acceptance. Despite initial controversies, OAGB accounted for 4.3% of all procedures performed (including primary surgery and revisions) in the latest IFSO report [11]. ASMBS data indicates that approximately 1,057 OAGB procedures were performed in the United States in 2020, increasing to 1,149 in 2021 and 1,338 in 2022 [12]. In light of that growing clinical use of OAGB, IFSO [13] and ASMBS [2] have issued formal position statements to clarify indications, surgical technique, and long-term outcomes. These developments underscore the need to synthesize current evidence, particularly in light of the expanding variability in procedural adaptations and reported results. Differences in pouch configuration, length of the biliopancreatic limb, and the use of anti-reflux sutures can influence surgical outcomes, including weight loss, GERD, and malnutrition. Recent analyses suggest that these technical factors should be carefully considered in patient selection and surgical planning [14, 15].

Given the sparse availability of evidence synthesis reports on key outcomes which limit the acceptance of OAGB, we aimed to perform a scoping review of the literature of OAGB studies focusing on four critical aspects:

1. Assessment of percentage of excess weight loss (%EWL).
2. Remission of metabolic and cardiovascular associated medical problems.
3. Postoperative complications.
4. Incidence and severity of gastroesophageal reflux (GERD).

## Methods

This scoping review is reported in compliance with PRISMA-ScR [16]. We used the PCC research question framework to construct our search strategy as:

- Population (P): adults of both sexes living with obesity;
- Concept (C): weight loss, remission of associated medical problems, complications, and GERD; and.
- Context (C): OAGB compared to other MBS modalities.

The key inclusion criteria for this scoping review of the literature were:

1. Studies in patients undergoing OAGB.
2. Studies reporting at least one of the following outcomes: weight loss, remission of associated medical problems (such as type 2 diabetes, clinical obesity or arterial hypertension), complications associated with OAGB MBS, and incidence of GERD after OAGB.
3. Study designs such as systematic reviews (with or without meta-analysis), clinical trials (randomized or non-randomized) and observational studies (cohort, case-control, or cross-sectional).

Case reports, narrative literature reviews, case series, studies for which the full text could not be retrieved, and retracted publications were excluded.

We queried four databases on 28 May 2025: MEDLINE (via PubMed and Embase), PubMed Central (Pubmed), Embase, and Web of Science (WoS). The reference search query was developed in PubMed by using medical subject headings (MeSH) and entry terms related to MBS modalities, combined with Boolean connectors “AND” and “OR”. The filters “clinicaltrial[Filter]”, “controlledclinicaltrial[Filter]”, “meta-analysis[Filter]”, “observationalstudy[Filter]”, “systematicreview[Filter]” and “humans[Filter]” were added to restrict the results. In addition, pediatric and pregnant populations were excluded by means of the Boolean connector “NOT”. There were no year of publication or language restrictions (as long as title and abstract were available in English). The full search queries per database (developed and validated by JMG and JAGD) are provided in the Supplementary Methods.

After the initial search, results were deduplicated with the Automated Systematic Search Deduplicator (ASySD) [17] in R version 4.5.0. The Active Learning for Systematic Reviews (ASReview) open-source software [18] was used to optimize screening. A set of pre-selected studies that covered all the outcomes of interest and selection criteria were used as prior knowledge. The ASReview training and configuration process is described in the Supplementary Methods and prior knowledge in Supplementary Table 1.

Three authors were involved in the initial screening of the studies (MAGG, AIGB and JAGD) and jointly assessed titles and abstracts, selecting those most relevant for inclusion in the different sections of the review by consensus. After completion of the initial screening with ASReview, two experienced authors in MBS (MAGG and AIGB) assessed full texts for selection. All systematic reviews and meta-analyses were included, whereas the selection of randomized controlled trials and observational studies was restricted to those not previously included in prior systematic reviews (novel studies) or considered as key studies by these two reviewers.

### Data extraction

Data were extracted by an experienced MBS surgeon (AIGB) and a nutritional scientist and biostatistician (AKG). Weight loss efficacy data were obtained based on different indicators. The main one was %EWL, according to the definition in the recommendations of Standardized Outcomes Reporting in MBS: [19]

$$\%EWL=\frac{W_0-W_t}{W_0-W_i}\times100$$

Where baseline weight is represented as $\:W_0$; postoperative weight ($W_t$); and ideal weight (the weight needed for a BMI = 25), $W_i$. We recorded the use of this definition in the studies and any deviations from it. The analysis also included additional efficacy outcomes such as the reduction in BMI, the percentage of total weight loss (%TWL), and the percentage of excess BMI loss, defined as the percentage reduction in BMI points above 25 units.

We report remission rates for noncommunicable diseases such as type 2 diabetes, arterial hypertension, dyslipidemia, and obstructive sleep apnea (OSA) in patients undergoing OAGB. Comparisons against other types of surgery were registered when available. Factors that influence the sustainability of long-term remission and its relationship with the duration of the disease before surgery were also noted. The following definitions were used per disease: [19]

- Remission of high blood pressure: blood pressure < 120/80 mmHg when off medication.
- Remission of type 2 diabetes mellitus: HbA1c < 6.0% without pharmacological treatment.
- Remission of dyslipidemia: triglycerides < 150 mg/dl and HDL cholesterol > 60 mg/dl without lipid-lowering therapy.

Postoperative complications were defined as those associated with bariatric intervention and were classified according to their time of onset: [19]

- Early complications: those that occur during surgery or within the first 30 days after the procedure.
- Late complications: those that occur after 30 days postsurgery.

We obtained the severity of GERD as reported in the studies and noted the method of assessment: patient self-reporting, endoscopy, or another type of study.

We grouped the results of the included studies in a table according to the main outcomes evaluated: (1) weight loss, (2) remission of associated medical problems (such as type 2 diabetes, clinical obesity or hypertension), (3) postoperative complications, and (4) GERD. For each study included in this review, we summarized the type of study design, the year of publication, as well as the lead author, and the overall findings reported. The evidence collected is presented in narrative format and supplemented with tables.

### Risk of bias assessment

We conducted a risk of bias assessment for the novel randomized controlled trials not included and assessed in prior systematic literature reviews, or which were considered key trials by at least three authors (JEOG, AIGB, and AKG). One reviewer (AKG) applied the Cochrane Risk of Bias 2.0 (RoB 2.0) tool [20] using the Excel tool to implement RoB 2 [21]. This was done to provide readers with additional information about the methodological quality of the experimental studies and to support more informed interpretation of novel and key studies.

## Results

**Fig. 1:**
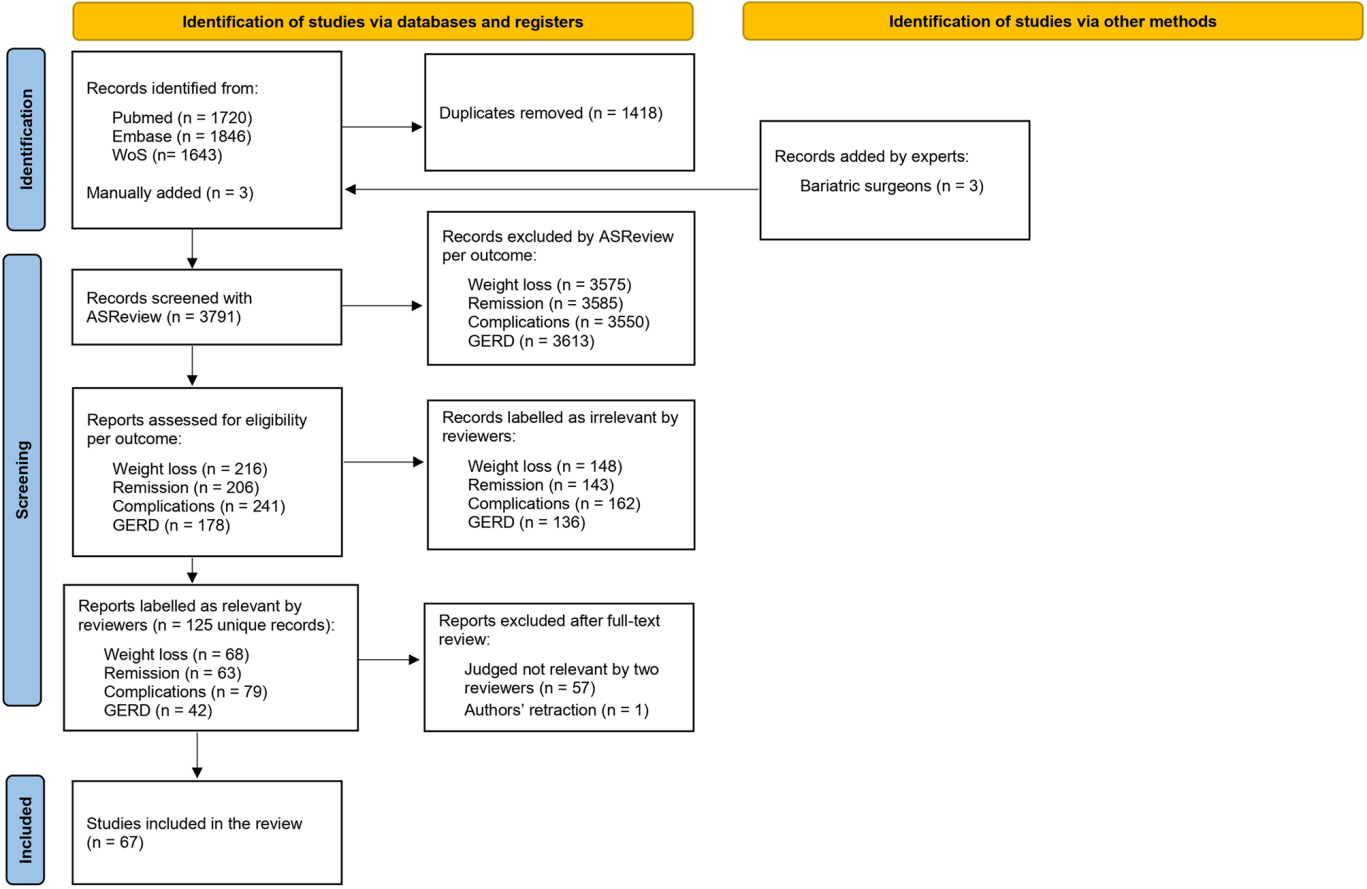
PRISMA Flowchart: study selection and exclusion

A total of 5209 records were identified in PubMed (*n* = 1720), Embase (*n* = 1846) and WoS (*n* = 1643). After deduplication, a total of 3791 records remained, which were screened with ASReview. A PRISMA-ScR flowchart with the total number of studies screened and the final number of studies included is shown in Fig. 1. Out of the total 67 studies included, 39 were systematic reviews (Supplementary Table 2), 12 randomized clinical trials (Supplementary Table 3), and 16 observational studies (Supplementary Table 4).

Based on the conducted search, one aim was to synthesize the available evidence on clinical outcomes frequently reported in comparative studies of OAGB versus SG, SADI-S, and RYGB. The most commonly reported outcomes were %EWL, %TWL, and BMI reduction. These outcomes were consistently reported at various postoperative time points, typically between 1 and 5 years, and in a few cases, up to 7 or 10 years. In nearly all studies: 42 in total (31 systematic reviews and 11 randomized controlled trials) OAGB was found to result in greater weight loss during the first 12 months. However, by 5 years, BMI reduction appeared to be similar between OAGB and RYGB.

### Weight loss outcomes with OAGB versus other bariatric procedures

Data from multiple meta-analyses and comparative studies suggest that OAGB is associated with greater weight loss compared to sleeve gastrectomy (SG), RYGB, and SADI-S, particularly in the short- and mid-term.

In terms of BMI reduction (Table 1), 4 out of 6 studies comparing OAGB with SG or RYGB reported greater BMI reduction in favor of OAGB at time points ranging from 6 months to 5 years. For instance, Li et al. [22] reported a mean BMI reduction difference of − 2.97 [95% CI: −3.33, − 2.60] at 2 years in favor of OAGB, while Onzi et al. [23] found a difference of − 1.78 [− 2.86, − 0.70] at 5 years when compared to SG. In contrast, Cosentino (2021) [24] and Luca (2023) [25] found no statistically significant differences at 2–3 years. Only one study (Jia 2020) [26] reported markedly higher BMI in the OAGB group versus RYGB, but results appeared inconsistent and likely influenced by baseline differences.

**Table 1:** BMI reduction in patients undergoing OAGB vs. other MBS

Author and publication date	Experimental	Control	Mean differences [95%CI] at different time points
Onzi TR 2024 [23]	OAGB	Other procedures	6 months: −1.39 [−3.77, 0.99] 1 year: −0.69 [−2.85, −0.70] 5 years: −1.78 [−2.86, −0.70]
Luca 2023 [25]	RYGB OAGB	OAGB SG	2–5 years: −0.88 [−1.80, 0.04] 2–5 years: −0.94 [−3.29, 1.41]
El-Gohary 2023 [27]	RYGB	OAGB	SMD in each comparison: 3–6 months: −0.53 [−1.48, 0.43] 1 year: 0.23 [0.02, 0.45] 2 years: 0.33 [0.15, 0.51] 3 years: −0.14 [−1.06, 0.78]
Li X 2023 [28]	OAGB	RYGB/SG	6 months: −0.74 [−2.98, 1.51] 1 year: −2.03 [−3.63, −0.44] 2 years: −2.97 [−3.33, −2.60] 5 years: −3.02 [−6.64, 0.61] % Excess of BMI loss 2 years: 9.89 [−4.79, 24.58]
Tasdighi 2022 [29]	OAGB 200 cm	OAGB 150 cm	1–2 years: −15.6 [−16.59, −14.61]
Cosentino 2021 [24]	SG	OAGB	3 years: −0.01 [−0.20, 0.18]
Jia D 2020 [26]	OAGB	RYGB	% Excess of BMI loss 1 year: 14.18 [4.18, 24.17] 2 years: 10.22 [3.05, 17.40] 5 years: 17.10 [15.76, 18.44]

Mean differences (or standardized mean differences, SMD) between groups are presented at various follow-up time points.

*OAGB* One anastomosis gastric bypass; *SG* sleeve gastrectomy; *RYGB* Roux-en Y gastric bypass.

Regarding percentage of excess body weight loss (%EWL) (Table 2), 9 out of 13 studies showed higher %EWL with OAGB compared to comparators (RYGB, SG, or SADI-S), with consistent differences ranging from approximately 2 to 15% points at 1 to 5 years. For example, Ali et al. [30] found a difference of 14.25% [5.34, 23.15] in favor of OAGB at 5 years versus SG, while Barzin et al. [31] reported a difference of 12.47% [7.79, 17.14] after 1 year. Differences were smaller in comparisons with RYGB, such as Malczak (2025) [32], which reported a 5-year advantage of 8.28% [1.99, 14.3] for OAGB. In contrast, one study (Santoro 2024) [33] found no significant differences (SMD − 0.06 [− 1.09, 0.98]) at 1 year.

**Table 2:** Percentage of excess of body weight loss in patients undergoing OAGB vs. other MBS

Author and publication date	Experimental	Control	Mean differences [95%CI] at different time points
Malczak 2025 [32]	OAGB	RYGB	1–5 years: 8.28 [1.99, 14.3]
Kermansaravi 2024 [14]	OAGB	SG/RYGB	1 year: 1.68 [0.08, 3.28]; 5 years: 2.51 [0.67, 4.36]
Onzi TR 2024 [23]	OAGB	Other procedures	1 year: 6.92 [1.16, 12.69] 5 years: 4.78 [−2.86, 12.25]
Ahmed 2024 [34]	SADI	OAGB	1 year: 10.78 [3.90, 17.66]; 2 years: 12.34 [−1.10, 25.86]
Santoro 2024 [33]	OAGB	RYGB	1 year: SMD: −0.06 [−1.09, 0.98]
El-Gohary 2023 [27]	OAGB	RYGB	SMD for all comparisons 3–6 months: −0.37 [−0.91, 0.18]; 1 year: −0.71 [−1.13, −0.28]; 2 years: −0.30 [−1.53, 0.92]; 3 years: −0.33 [−0.52, 0.15]
Li X 2023 [28]	RYGB/SG	OAGB	6 months: 2.19 [−4.14, 8.51] 1 year: 3.55 [0.90, 6.19]
Ali M 2023 [30]	OAGB	SG	1 year: 8.03 [4.54, 11.52] 2 years: 8.94 [2.95, 14.94] 3 years: 8.93 [5.75, 12.10] 4 years: 15.09 [0.87, 29.31] 5 years: 14.25 [5.34, 23.15]
Balamurugan G 2023 [35]	RYGB	OAGB	1 year: 5.89 [−3.13, 14.91] 2 years: 0.52 [−10.71, 11.75] 3 years: 10.27 [0.67, 19.87] 5 years: 10.37 [1.03, 19.71]
Salman 2023 [36]	OAGB 200 cm	OAGB 150 cm	1–2 years: −1.01 [−5.96, 3.93]
Barzin M 2023 [31]	OAGB	SG	< 1 year: 6.13 [3.60, 8.65] > 1 year: 12.47 [7.79, 17.14]
Magouliotis 2019 [7]	OAGB	RYGB	1 year: −6.02 [−8.84, −3.20]; 2 years: −7.33 [−10.08, 5.53] 5 year: −12.82 [−20.27, −5.37]
Magouliotis 2017 [44]	OAGB	SG	1 year: −6.52 [−11.65, −1.40]; 2 years: −16.78 [−38.92, 5.37]

Mean differences (or standardized mean differences, SMD) between groups are presented at various follow-up time points.

*OAGB* One anastomosis gastric bypass; *SG* sleeve gastrectomy; *RYGB* Roux-en Y gastric bypass; *SADI* Single Anastomosis Duodeno-Ileal bypass.

In the analysis of % total body weight loss (%TBWL) (Table 3), most comparative studies favored OAGB, especially in comparisons with SG and SADI-S. For instance, Barzin et al. [31] reported a mean difference of 7.95% [5.69, 10.21] after more than 1 year when comparing OAGB to SG, and Ahmed et al. [37] found a 5.32% [0.96, 9.68] difference at 2 years compared to SADI-S. Differences were smaller or non-significant in RYGB comparisons, such as Ahmed (2025), which showed no significant differences at 1 and 2 years.

Overall, OAGB was favored in approximately 75% of the included comparisons, particularly in early postoperative periods (1–2 years). However, differences tended to narrow at 5 years, especially when compared to RYGB. Across outcomes, variation in surgical technique (e.g., limb length), definition of weight loss metrics, and follow-up time frames must be considered when interpreting these findings.

**Table 3:** Percentage of total weight loss in patients undergoing OAGB vs. other MBS

Author and publication date	Experimental	Control	Mean differences [95%CI] at different time points
Ahmed 2025 [37]	OAGB	RYGB	6 months: 1.87 [0.80, 2.94] 1 year: −0.37 [−1.51,0.77] 2 years: −1.30 [−4.59, 2.00] 3 years: −1.54 [−5.32, 2.25]
Malczak 2025 [32]	RYGB	OAGB	1 year: 4.10 [−1.01, 9.52]
Ahmed 2024 [34]	SADI	OAGB	1 year: 4.30 [2.43, 6.17] 2 years: 5.32 [0.96, 9.68]
Balamurugan G 2023 [35]	RYGB	OAGB	1 year: 3.41 [−1.63, 8.45] 2 years: 1.21 [−4.18, 6.60] 3 years: 6.37 [2.25, 10.49] 5 years: 5.79 [1.93, 13.51]
Salman 2023 [38]	OAGB 200 cm	OAGB 150 cm	1–2 years:−2.61 [−4.55, −0.66]
Barzin M 2023 [31]	OAGB	SG	< 1 year: 5.34 [3.2, 7.47] > 1 year: 7.95 [5.69, 10.21]
Li Y 2022 [47]	OAGB 150 cm	OAGB 200 cm	1 year: −1.57 [−2.46, −0,68]

Mean differences between groups are presented at various follow-up time points.

*OAGB* One anastomosis gastric bypass; *SG* sleeve gastrectomy; *RYGB* Roux-en Y gastric bypass; *SADI* Single Anastomosis Duodeno-Ileal bypass.

#### Influence of biliopancreatic loop length (BPL)

Differences in biliopancreatic limb (BPL) length within OAGB procedures appear to influence weight loss outcomes, particularly in terms of total weight and BMI reduction. The comparison between standard (150 cm) and extended (200 cm) limb lengths suggests that longer BPLs may enhance the malabsorptive effect of the surgery, contributing to greater weight loss during the first postoperative years. These differences are more evident in outcomes such as BMI and %TBWL than in %EWL, where the impact of limb length seems less consistent. This variability may be due to the lack of standardization in how excess weight is defined and measured across studies. Moreover, while longer BPLs may offer modest improvements in metabolic efficacy, they must be weighed against the potential for increased nutritional risk, particularly in long-term follow-up [22–24]. These findings highlight the importance of individualized surgical planning and reinforce the need for standardized reporting of surgical techniques in future research to allow more accurate comparisons.

### Remission of associated medical problems

The available evidence suggests that OAGB is associated with favorable outcomes in type 2 diabetes remission when compared to various metabolic and bariatric procedures, including sleeve gastrectomy (SG), Roux-en-Y gastric bypass (RYGB), biliopancreatic diversion (BPD), and standard medical therapy (Table 4). Across the included studies, OAGB consistently showed non-inferior and, in several cases, superior remission rates, particularly in comparisons with SG and conservative treatments. However, direct comparisons with RYGB and BPD yielded mixed results, with some studies favoring OAGB and others favoring the comparator procedure.

**Table 4:** Remission of type 2 diabetes in patients undergoing OAGB vs. other MBS

Author and publication date	Experimental	Control	OR or RR and 95%CI	Definition of remision
Zang 2025 [39]	OAGB	NTS	Total: 10.28 [1.87, 56.40] Partial: 11.24 [3.44, 36.70]	Complete diabetes remission was defined as having a HbA1c Level below 6.0% (42 mmol/mol) and a fasting glucose Level below 100 mg/dL (5.6 mmol/L), measured at least 12 months after stopping glucose-lowering medication. Partial diabetes remission was defined as having a HbA1c Level below 6.5% (42 mmol/mol) and a fasting glucose Level below 100–125 mg/dL (5.6–6.9 mmol/L) measured at least 12 months.
Kermansaravi 2024 [14]	OAGB	SG/RYGB	1 year: 1.20 [0.94, 1.53] 5 years: 1.28 [1.10, 1.49]	Not specified
Onzi TR 2024 [23]	OAGB	RYGB/SG	1.03 [0.87, 1.22]	Not specified
Ali 2023 [30]	OAGB	SG	1 year: 1.80 [1.28, 2.53] 2 years: 2.25 [1.37, 3.70] 5 years: 1.16 [0.77, 1.74]	Not specified
Li X 2023 [28]	OAGB	RYGB	1.11 [0.99, 1.26]	Not specified
Salman 2023 [38]	OAGB 200 cm	OAGB 150 cm	0.76 [0.37, 1.57]	Not specified
Barzin M 2023 [31]	OAGB	SG	0.77 [0.28, 2.16]	Not specified
Carmona 2021 [40]	OAGB	VBG	0.53 [0.04, 6.53]	Not specified
		LDS	0.83 [0.40, 1.71]	
		LRYGB	0.68 [0.37, 1.25]	
		LSG	0.55 [0.31, 0.96]	
		LAGB	0.34 [0.06, 2.02]	
		BPD	0.27 [0.01, 8.60]	
		MT	0.14 [0.04, 0.49]	
Cresci 2020 [41]	SG	OAGB	2.29 [1.10, 4.75]	HbA1c < 48 mmol/mol with no drug therapy
Ding L [22]	BPD	OAGB	0.75 [0.05, 11.58]	HbA1c levels < 6.0% at consecutive annual clinic visits with no use of anti-hyperglycaemic medications oe as individual studies.
	GCP		0.02 [0.00, 0.29]	
	LAGB		0.05 [0.00, 0.43]	
	SG		0.20 [0.03, 1.29]	
	RYGB		0.19 [0.03, 1.34]	
Park 2019 [42]	OAGB	Standard of care	12.21 [4.74, 31.45]	Not specified
Magouliotis 2019 [7]	OAGB	RYGB	0.41 [0.25, 0.69	Not specified
Kodama 2018 [43]	BPD	OAGB	1.99 [0.42, 9.51]	Achievement of glycemia below the diabetic range in the absence of active pharmacologic therapy
	BPD/DS		1.32 [0.63, 2.80]	
	RYGBP		1.64 [0.99, 2.71]	
	SG		1.85 [1.15, 2.97]	
	LAGB		4.34 [1.69, 11.15]	
	GCP		4.69 [1.73,12.71]	
	DJB		15.28 [0.86, 270.05]	
	NST		22.91 [9.18, 57.17]	
Magouliotis 2017 [44]	OAGB	SG	0.46 [0.32, 0.64]	Not specified

Data in Carmona 2021 are RR.

*BPD* biliopancreatic diversion; *DJB* duodenal-jejunal bypass; *DS* duodenal switch; *OAGB* One anastomosis gastric bypass; *GCP* greater curvature plication; *LAGB* laparoscopic adjustable gastric banding; *SG* sleeve gastrectomy; *RYGB* Roux-en Y gastric bypass; *NST* non-surgical treatment

One of the main challenges in interpreting these findings lies in the lack of standardized definitions for diabetes remission. Only a minority of studies clearly defined remission criteria, typically based on HbA1c thresholds and the discontinuation of glucose-lowering medications. In most cases, the absence of explicit definitions limited the comparability of results. Furthermore, variations in follow-up duration, surgical technique (e.g., BPL length), and baseline metabolic control may influence the observed remission rates.

Overall, while the data indicate a potential advantage of OAGB for glycemic improvement, the heterogeneity in study design and outcome definitions underscores the need for standardized reporting and high-quality comparative trials to better understand the long-term metabolic effects of OAGB.

Regarding the remission of dyslipidemia (Table 5) the available data on dyslipidemia remission after OAGB are limited but suggest a potential benefit compared to other bariatric procedures. In comparisons with sleeve gastrectomy (SG), results were inconsistent: Ali et al. [30] reported markedly higher remission rates with OAGB across 1 to 5 years (ORs ranging from 5.20 to 14.44), whereas Magouliotis et al. (2017) [44] found significantly better outcomes with SG (OR 0.32 [0.19–0.56]). Comparisons with RYGB, however, were inconsistent, with one study showing a modest advantage for OAGB and others showing either no difference or a tendency favoring the comparator. Notably, none of the included studies explicitly defined what constituted remission of dyslipidemia, making interpretation of effect sizes more challenging. The variability in results likely reflects heterogeneity in baseline lipid profiles, pharmacologic management, and duration of follow-up.

**Table 5:** Remission of dyslipidemia by OAGB vs. other MBS

Author and publication date	Experimental	Control	OR or RR and 95%CI	Definition of remision
Ali 2023 [30]	OAGB	SG	1 year: 5.20 [4.12, 6.55] 2 years: 5.62 [4.48, 7.05] 5 years: 14.44 [11.03, 18.05]	Not specified
Li X 2023 [28]	OAGB	RYGB	1.39 [1.20, 1.61]	Not specified
Magouliotis 2019 [7]	OAGB	RYGB	0.55 [0.16, 1.91]	Not specified
Magouliotis 2017 [44]	OAGB	SG	0.32 [0.19, 0.56]	Not specified

*OAGB* One anastomosis gastric bypass; *SG* sleeve gastrectomy; *RYGB* Roux-en Y gastric bypass.

Arterial hypertension also showed a remission rate of 50–66.7% in patients undergoing OAGB [45]. One systematic review with meta-analysis confirmed that hypertension remission was 64.3% in OAGB, comparable to RYGB. These results reinforce the hypothesis that weight loss and reduction of systemic inflammation play a key role in improving blood pressure in patients with obesity [35]. 

OSA showed a remission rate of 97% in patients with OAGB, with no significant differences to RYGB. The substantial weight loss associated with MBS contributes to decreased upper airway resistance, improving sleep quality and reducing reliance on continuous positive airway pressure (CPAP) devices [9]. 

About another remission of medical conditions or incidence of complications (Table 6). Overall, in comparisons with RYGB, OAGB demonstrated a higher likelihood of gastroesophageal reflux disease (GERD) incidence and de novo onset, along with a lower probability of GERD remission, suggesting a less favorable profile for reflux-related outcomes. When compared to SG, OAGB showed potential advantages in the resolution of hypertension and osteoarthritis at short-term follow-up, although an increased risk of obstructive sleep apnea could not be ruled out. No consistent differences were observed in hypertension outcomes between OAGB and RYGB, nor between variations in OAGB limb length. In comparisons with LSG, OAGB appeared to offer improved outcomes in blood pressure control. Collectively, these findings suggest that OAGB may provide benefits in metabolic disease remission, particularly for hypertension, but may be associated with an elevated risk of GERD-related complications.

**Table 6:** Risk of remission or incidence of other associated medical problems by OAGB vs. other MBS

Author and publication date	Control	Experimental	Diseases	OR or RR and 95%CI	Definition
Kapellas 2024 [46]	OAGB	RYGB	GERD incidence	3.14 [1.23, 8.03];	Not specified
			GERD remission	0.32 [0.06, 1.77]	Not specified
			GERD *de novo*	5.65 [1.53, 20.82]	Not specified
			Endoscopically proven esophageal reflux disease	1.63 [0.58, 4.58]	Not specified
Ali 2023 [30]	SG	OAGB	HT	1 year: 0.76 [0.62, 0.94] 2 years: 0.92 [0.75, 1.13] 5 years: 0.83 [0.68, 1.00]	Not specified
			OSAS	1 year: 3.92 [0.92, 16.76]	Not specified
			OA	1 year: 0.55 [0.31, 0.97]	Not specified
Li X 2023 [28]	OAGB	RYGB	HT	1.10 [0.94, 1.29]	Not specified
			GERD	0.35 [0.11, 1.18]	Not specified
Salman 2023 [38]	OAGB 150 cm	OAGB 200 cm	HT	1.21 [0.73, 1.99]	Not specified
Barzin M 2023 [31]	OAGB	SG	HT	1.63 [1.06, 2.50]	Not specified
Magouliotis 2019 [7]	OAGB	RYGB	HT	0.93 [0.55, 1.56]	Not specified
Magouliotis 2017 [44]	OAGB	SG	HT	0.67 [0.49, 0.90]	Not specified

*SG* Sleeve gastrectomy; *RYGB* Roux-en Y gastric bypass; *OAGB* One anastomosis gastric bypass. *GERD* Gastroesophageal Reflux Disease; *HT* Hypertension. *OSAS* Obstructive Sleep Apnea Syndrome.

The available evidence on postoperative complications (Table 7) following OAGB reveals a mixed pattern, with certain adverse events being more frequent compared to other metabolic and bariatric procedures, while others are less common. Several studies comparing OAGB with RYGB or SG reported higher odds of marginal ulcers, malnutrition, and new-onset gastroesophageal reflux disease (GERD) after OAGB. Conversely, OAGB was associated with a lower risk of postoperative bile reflux and anastomotic leakage in some comparisons. However, complication definitions were not standardized across studies, and many outcomes were reported in aggregated form (e.g., “major surgical complications” or “unspecified adverse events”), limiting the precision of comparisons. Nutritional deficiencies (including hypoalbuminemia, low ferritin, and vitamin deficiencies) were assessed in comparisons of different BPL lengths in OAGB, but results were inconsistent and often non-significant. Overall, the heterogeneity in outcome reporting and definitions underscores the need for standardized criteria in future studies to better assess and compare the safety profiles of OAGB and other surgical techniques.

**Table 7:** Risk of medical complications by OAGB vs. other MBS

Author and publication date	Experimental	Control	Complication	Outcome	Definition
Ahmed 2025 [37]	OAGB	RYGB	GERD novo	2.58 [1.55, 4.30]	Not specified
			Incidence of marginal ulcers	2.70 [1.07, 6.84]	Not specified
Zang 2025 [39]	OAGB	NTS	Surgical and GI complications (unspecified)	1.08 [0.24, 4.89]	Not specified
			Macrovascular	2.45 [0.11, 53.87]	Not specified
			Microvascular	1.04 [0.16, 6.62]	Not specified
Ahmed 2024 [34]	SADI	OAGB	Postoperative biliary reflux	0.15 [0.04, 0.53]	Not specified
			Incidence of anastomotic ulcers postoperatively	0.23 [0.05, 1.10]	Not specified
Santoro 2024 [33]	OAGB	RYGB	OAGB anastomotic leak	−0.00 [−0.02, −0.02]	Not specified
			RYGB post-op reflux	1.82 [1.23, 2.69]	Not specified
			OAGB post-op bleeding	−0.01 [−0.03, 0.01]	Not specified
Luca 2023 [25]	RYGB	OAGB	Unspecified adverse events	0.86 [0.15, 5.08].	Not specified
	SG		Unspecified adverse events	1.95 [0.53, 7.22].	Not specified
El-Gohary 2023 [27]	OAGB	RYGB	Major surgical complications (unspecified)	1.57 [0.80, 3.07]	Not specified
			Anastomotic leak	1.08 [0.32, 3.62]	Not specified
			Major bleeding	2.20 [0.47, 10.32]	Not specified
Ali 2023 [30]	OAGB	SG	Overall complications	1.04 [0.87, 1.25]	Not specified
			Leakage	0.38 [0.19, 0.79]	Not specified
			GERD	0.52 [0.20, 1.35]	Not specified
			Ulcer	6.21 [2.52, 15.32]	Not specified
			Malnutrition	4.71 [2.63, 8.45]	Not specified
Li X 2023 [28]	RYGB	OAGB	Intraoperative complications	2.31 [0.78, 6.79]	Not specified
			Early postoperative complications	0.45 [0.21, 0.97]	Not specified
			Late postoperative complications	0.91 [0.61, 1.35]	Not specified
Salman 2023 [38]	OAGB 200 cm	OAGB 150 cm	Hypoalbuminemia	0.91 [0.39, 2.13]	Not specified
			Low protein levels	0.60 [0.20, 1.86]	Not specified
			Low ferritin levels	0.66 [0.37, 1.18]	Not specified
			Low vitamin B12	0.76 [0.21, 2.76]	Not specified
			Low folate levels	0.73 [0.34, 1.58]	Not specified
			Low vitamin D levels	0.93 [0.42, 2.05]	Not specified
Li Y 2022 [47]	200 cm BPL	Short BPL	Ferritin deficiency	0.82 [0.51, 1.31]	Not specified
			Iron deficiency	0.87 [0.44, 1.74]	Not specified
			Hypoalbuminemia	0.14 [0.02, 1.11]	Not specified
			Vitamin D deficiency	0.96 [0.62, 1.49]	Not specified
			Vitamin B12 deficiency	1.42 [0.69, 2.95]	Not specified
			Leakage	0.55 [0.09, 3.27]	Not specified
Magouliotis 2019 [7]	OAGB	RYGB	Leaks	1.33 [0.62, 2.84]	Not specified
			Malnutrition	0.12 [0.04, 0.41]	Albumin concentration of less than 30 g/L or prealbumin concentration of less than 0.2 g/L, or both.
			Marginal Ulcer	0.74 [0.35, 1.57]	Not specified
			Intra-abdominal bleed	1.61 [0.62, 4.13]	Not specified
			Internal hernia	5.26 [1.76, 15.69]	Not specified
			Dumping	0.77 [0.33, 1.80]	Not specified
			Bowel Obstruction	3.75 [1.42, 9.92]	Not specified

*BPD* biliopancreatic diversion; *DJB* duodenal-jejunal bypass; *DS* duodenal switch; *OAGB* One anastomosis gastric bypass; *GCP* greater curvature plication; *LAGB* laparoscopic adjustable gastric banding; *SG* sleeve gastrectomy; *RYGB* Roux-en Y gastric bypass; *NST* non-surgical treatment

### Gastroesophageal reflux disease and bile reflux

Both GERD and bile reflux can significantly impact quality of life and lead to long-term complications. Current evidence shows that these adverse effects are not only frequent, but may also be underestimated due to its difficult diagnosis and variability in the criteria used per study. Reported incidences of *de novo* GERD after OAGB range between 6% and 57% [48], depending on follow-up time and the diagnostic method used. In the YOMEGA randomized controlled study, a rate of 41% in OAGB vs. 18% in RYGB was reported, suggesting an increased risk of reflux symptoms in patients undergoing OAGB. It has been observed that 8% of patients with severe GERD after OAGB require conversion to RYGB, indicating that in certain cases revision surgery is the only effective solution [9, 49]. 

Nonetheless, some studies have also reported that OAGB can improve pre-existing reflux in patients with mild to moderate GERD, with a reduction similar to that observed in RYGB in terms of 24-hour pH-metry and endoscopy [50, 51]. 

Recent evidence suggests that the presence of a hiatal hernia is associated with an increased risk of de novo GERD after OAGB [52]. Consequently, the identification and repair of hiatal hernias during OAGB may be beneficial in reducing postoperative reflux symptoms. This strategy is especially recommended for patients with significant preoperative reflux or large hiatal defects [52]. 

Bile reflux is another relevant adverse effects of OAGB, since the absence of a second anastomosis allows direct exposure of the gastric and esophageal mucosa to bile acids. Studies have reported that 31.6% of patients have bile reflux in scintigraphy studies, while endoscopy has revealed bile related inflammatory signs in 39.5% of cases. Only 2.6% report bile reflux in the esophagus, but the lack of long-term studies makes it impossible to determine whether this phenomenon could increase the risk of premalignant lesions [53]. 

The diagnosis of bile reflux is complex, as its symptoms can overlap with those of acid reflux. 24-hour pH monitoring studies have identified post-OAGB GERD as a combination of acid reflux (30.2%), bile reflux (27.9%), and mixed (11.6%), suggesting that clinical symptoms may be insufficient to differentiate the two processes.

#### Anti-reflux sutures

Some studies have evaluated the efficacy of adding anti-reflux sutures to reduce the incidence of *de novo* GERD. One randomized controlled study reported that this modification reduced the incidence of GERD in 16.1–6.2%, although without reaching statistical significance [50]. 

#### Conversion to RYGB

In patients with severe reflux, conversion to RYGB has proven to be an effective strategy. Approximately 1.6% of OAGB patients require revision surgery for GERD, being the conversion to RYGB the most frequent option in 91.7% of cases [26]. 

#### Endoscopic Follow-Up

Endoscopic monitoring is used for the early detection of esophagitis, marginal ulcers, or signs of chronic bile exposure, which could predispose to premalignant changes. In patients with persistent symptoms, evaluation with endoscopy and pH testing is recommended to determine the severity of reflux.

These findings reinforce the importance of a careful patient selection, prioritizing OAGB in those without a history of severe reflux and avoiding its use in patients with advanced esophagitis or Barrett’s esophagus diagnosis [54]. 

### Risk of bias

**Fig. 2:**
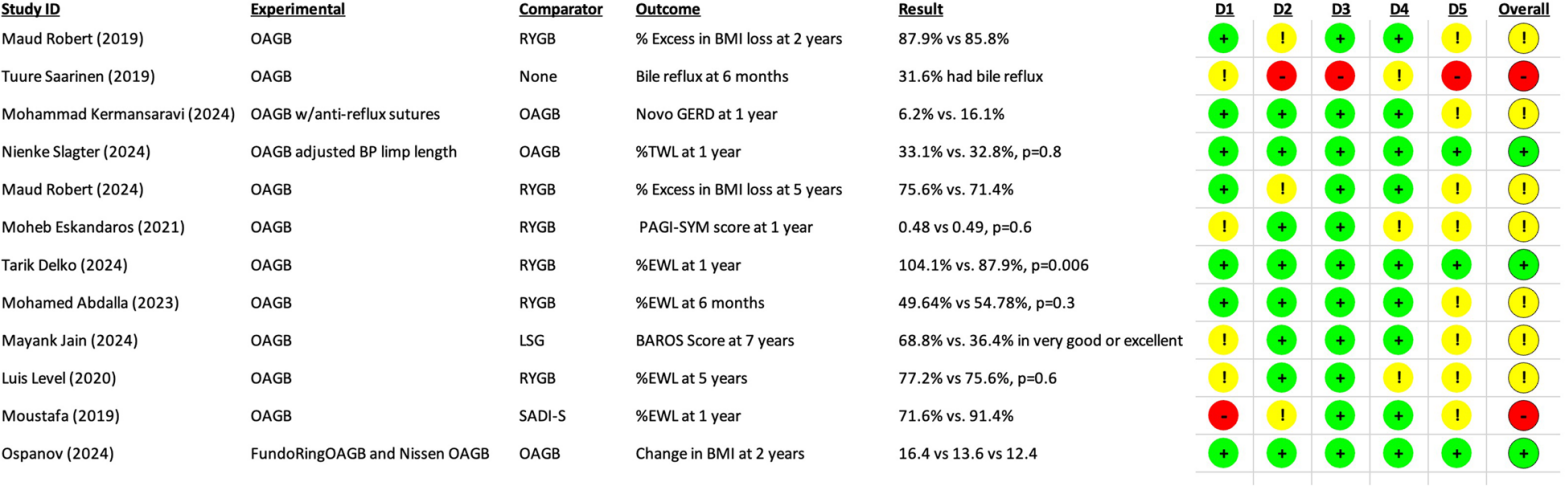
Risk of bias assessment and key outcomes of novel andkey randomized controlled trials (RCT) included in the scoping review. Each row represents an RCT evaluating one-anastomosis gastric bypass (OAGB) with different comparators and outcomes. The results column summarizes the main finding reported by each study, while the color-coded domains (D1–D5) reflect the RoB 2.0 assessment: randomization process (D1), deviations from intended interventions (D2), missing outcome data (D3), measurement of the outcome (D4), and selection of the reported result (D5). Green = low risk, yellow = some concerns, red = high risk. The overall risk of bias judgment is shown in the last column.

A total of 11 randomized controlled trials evaluating OAGB were assessed using the Cochrane RoB 2.0 tool (Fig. 2). Most trials reported favorable outcomes in terms of weight loss, comorbidity improvement, and patient-reported measures when compared to standard procedures such as RYGB, SADI-S, or LSG. However, the risk of bias varied across studies. Domains related to missing outcome data (D3) and deviations from intended interventions (D2) showed the highest frequency of “some concerns” or “high risk” judgments. One study (Tuure Saarinen, 2019) was classified as having high risk of bias overall, primarily due to lack of control group. The remaining studies were rated as either low risk or having some concerns, with variability across domains. The heterogeneity in outcome definitions (e.g., GERD, %EWL, quality of life scores) and follow-up duration was notable. Full details of RoB judgments and reported outcomes are presented in Fig. 2. These findings provide context on the methodological rigor of recent RCTs examining OAGB, but may not be representative of the studies previously assessed in other systematic reviews of OAGB.

## Discussion

The findings of this review show that OAGB is a highly effective procedure in terms of weight loss and remission of metabolic medical problems. In the short and long term, OAGB shows a %EWL greater than that of RYGB and SG, with values reaching 84.27% per year and remaining at 67.5% even after a decade [11, 14, 37]. Metabolic remission of type 2 diabetes ranges from 76.8 to 100%. In some studies, OAGB demonstrated higher remission rates than SG or conservative treatments, and occasionally superior outcomes compared to RYGB [32, 39]. However, other analyses reported comparable or even lower remission rates than RYGB [7, 44]. Hypertension, dyslipidemia, and obstructive sleep apnea also show significant improvements. However, the length of the biliopancreatic loop plays a crucial role in the outcome [8, 15], as lengths greater than 200 cm can optimize weight loss at the cost of an increased risk of nutritional deficiencies [14, 32]. 

It is important to interpret the findings of this review in light of the variability in the strength and quality of the available evidence. While meta-analyses and randomized controlled trials (RCTs) were prioritized for data synthesis, considerable heterogeneity remains in terms of study design, follow-up duration, surgical technique, and outcome definitions. Although the outcomes reported (such as excess weight loss, associated medical problems remission, and complication rates) are familiar to bariatric surgeons and researchers, few studies clearly define these endpoints. While this may seem self-evident, transparent outcome reporting is essential for scientific reproducibility. For instance, while %TWL and BMI reduction are typically reported as relative changes from baseline, %EWL is inconsistently defined across studies. Most publications fail to specify how ideal weight was calculated or which formula was applied. Even highly cited RCTs in the field of OAGB differ in how they define ideal weight or excess weight between protocols and final publications [9, 55]. Although IFSO defines a healthy weight range as corresponding to a BMI of 18.5–25 kg/m², it does not specify a standard method for ideal weight calculation in the context of %EWL estimation [12]. This lack of consistency presents a major limitation, as it may bias pooled estimates in meta-analyses when studies with different underlying definitions are analyzed together. Moving forward, systematic reviews should consider reporting standardized mean differences (SMD) [56] instead of relying solely on %EWL values to enhance comparability. In our review, only two of the included systematic reviews reported SMD.

Additionally, we identified similar heterogeneity in the way of remission of associated medical problems and postoperative complications were defined and reported. While we included in the Methods section the standard definitions currently used in the literature for remission of type 2 diabetes, hypertension, and other associated medical problems, many studies deviated from these definitions or did not report them at all. In some cases, complications were aggregated into broad categories or binary variables, limiting interpretability. For this reason, we included in the results section the specific definitions or criteria used in each study to define efficacy and safety endpoints. This decision aimed to improve transparency and support a more critical and informed interpretation of the reported findings.

These nuances are further complicated by the potential for publication bias and selective reporting, particularly in single-center or retrospective observational studies. Acknowledging these limitations is crucial to avoid overinterpreting the strength of the evidence and to support more rigorous and transparent reporting in future MBS research.

Our scoping review presents methodological strengths. First, we followed a structured, and reproducible systematic search of studies. The ASReview tool was implemented to optimize the screening process, which made it possible to streamline and improve efficiency in the selection of studies. Using a set of pre-selected studies as background knowledge allowed us to train an artificial intelligence model able to identify relevant studies in a fraction of time compared to a conventional systematic review. The involvement of three independent reviewers in the initial screening of titles and abstracts and the evaluation of full texts by two review authors who are experts in MBS strengthened the selection of studies based on sound clinical criteria. Finally, by not restricting the search by language or date of publication, a diversity of studies that reflect the historical and global evolution of OAGB research is presented in this study.

However, this review also has some limitations. First, the exclusion of studies whose full text was not available may have omitted relevant studies. Second, as the objective of a scoping review was to map the evidence reported and not to summarize the evidence through a meta-analysis, we did not include detailed information on the characteristics of patients undergoing the various MBS procedures. Therefore, it is important for readers to consider that the synthesis and conclusions presented were made without accounting for patient-level variables, which may vary substantially across the different studies included in the meta-analyses. Likewise, the quality of the systematic reviews and meta-analyses summarized here was not assessed and we can therefore not conclude on the quality of the evidence they provide.

Although some studies report outcomes up to 10 years postoperatively, data beyond this timeframe remain scarce, limiting our understanding of very long-term outcomes and rare complications. Potential late issues such as revision surgery or malignancy related to chronic bile reflux are not yet well characterized for OAGB, underscoring the need for further long-term follow-up studies. Lastly, our study did not evaluate other dimensions which are primordial for decision-making such as quality-of-life outcomes, cost-effectiveness, or other comparisons of different surgical technique modifications of OAGB. Despite its limitations, this scoping review of the literature can inform clinical guideline development and decision-making across the clinical outcomes assessed here by providing a broad overview of the literature on four major clinical aspects of OAGB.

## Conclusion

OAGB is an effective MBS modality in terms of weight loss and remission of metabolic medical problems, with favorable outcome rates comparable to or superior to those of RYGB, but its association with GERD and bile reflux remains a significant concern. Although most cases can be managed with medication and modifications in surgical technique, a low percentage of patients develop severe symptoms that require revision surgery. Therefore, patient selection, personalization of surgical technique, and regular endoscopic follow-up are essential to minimize these risks and optimize long-term outcomes.

The limitations found are associated with the heterogeneity that exists in the OAGB technique, such as variations in the length of the biliopancreatic loop and the size of the gastric pouch in the different studies. Short follow-up periods and variability in GERD assessment methods affect associated medical problems remission outcomes and the efficacy and safety assessment of the procedure, especially on bile reflux-related esophageal changes, which is where the greatest concern about this procedure lies.

## Supplementary Information

Supplementary Material 1: Supplementary Methods.

Supplementary Material 2: Supplementary Tables 1 to 4.

## Data Availability

All data generated or analysed during this study are included in this published article and its supplementary information files. Additional datasets, code, and documentation used for data processing and analysis are openly available in the GitHub repository: [https://github.com/javimangal/OAGB-review](https:/github.com/javimangal/OAGB-review).

## Abbreviations

ASReview: Active Learning for Systematic Reviews
BAROS: Bariatric Analysis and Reporting Outcome System
BPL: Biliopancreatic Loop
BMI: Body Mass Index
CPAP: Continuous Positive Airway Pressure
GERD: Gastroesophageal Reflux Disease
GLP: Gastrojejunal Limb Length
HbA1c: Hemoglobin A1c
MeSH: Medical Subject Headings
MBS: Metabolic and Bariatric Surgery
OSA: Obstructive Sleep Apnea
OAGB: One Anastomosis Gastric Bypass
OS: Other Surgeries (e.g., SADI-S, LSG)
%EWL: Percentage of Excess Weight Loss
PCC: Population, Concept, Context
PRISMA-ScR: Preferred Reporting Items for Systematic Reviews and Meta-Analyses extension for Scoping Reviews
RYGB: Roux-en-Y Gastric Bypass
SADI-S: Single Anastomosis Duodeno-Ileal Bypass with Sleeve Gastrectomy
SG: Sleeve Gastrectomy
TSBL: Total Small Bowel Length
