# Supplementary material for: One anastomosis gastric bypass (OAGB): a scoping review
Source: BMC Surg. 2025 Oct 10;25:471. doi: 10.1186/s12893-025-03215-x (PMC12512307; doi:10.1186/s12893-025-03215-x)
Supplement: Supplementary file 1 — Supplementary Material 1: Supplementary Methods. [file 12893_2025_3215_MOESM1_ESM.docx]

Supplementary Methods

1. **Search strategy**

**Medline and PubMed Central – Pubmed**

| **Search number** | **Search Details** |
| --- | --- |
| 1 | "Bariatric Surgery"[MeSH Terms] |
| 2 | "Bariatric Surgery"[All Fields] |
| 3 | "obesity/surgery"[MeSH Terms] |
| 4 | "obesity, morbid/surgery"[MeSH Major Topic] |
| 5 | #1 OR #2 OR #3 OR #4 |
| 6 | "Gastric Bypass"[MeSH Terms] |
| 7 | "Gastric Bypass/methods"[MAJR] |
| 8 | "Gastric Bypass/adverse effects"[MAJR] |
| 9 | "One anastomosis gastric bypass"[All Fields] |
| 10 | "OAGB" [All Fields] |
| 11 | "Mini-gastric Bypass"[All Fields] |
| 12 | "MGB" [All Fields] |
| 13 | "omega loop gastric bypass" [All Fields] |
| 14 | "OLGB" [All Fields] |
| 15 | "Single anastomosis gastric bypass" [All Fields] |
| 16 | "SAGB" [All Fields] |
| 17 | #6 OR #7 OR #8 OR #9 OR #10 OR #11 OR #12 OR #13 OR #14 OR #15 OR #16 |
| 18 | #5 AND #17 |
| 19 | clinicaltrial[Filter] OR controlledclinicaltrial[Filter] OR meta-analysis[Filter] OR observationalstudy[Filter] OR systematicreview[Filter] |
| 20 | #18 AND #19 |
| 21 | humans[Filter] |
| 22 | #20 AND #21 NOT "Pregnant*" NOT "Child"[Mesh] NOT "Child*" |

**PubMed full search string (1720 results):**

(((("Bariatric Surgery"[MeSH Terms] OR "Bariatric Surgery"[All Fields] OR "obesity/surgery"[MeSH Terms] OR "obesity, morbid/surgery"[MeSH Major Topic]) AND ("Gastric Bypass"[MeSH Terms] OR "gastric bypass/methods"[MeSH Major Topic] OR "gastric bypass/adverse effects"[MeSH Major Topic] OR "One anastomosis gastric bypass"[All Fields] OR "OAGB"[All Fields] OR "Mini-gastric Bypass"[All Fields] OR "MGB"[All Fields] OR "omega loop gastric bypass"[All Fields] OR "OLGB"[All Fields] OR "Single anastomosis gastric bypass"[All Fields] OR "SAGB"[All Fields]) AND ("clinical trial"[Publication Type] OR "controlled clinical trial"[Publication Type] OR "meta analysis"[Publication Type] OR "observational study"[Publication Type] OR "systematic review"[Filter]) AND "humans"[MeSH Terms]) NOT "pregnant*"[All Fields]) NOT "Child"[MeSH Terms]) NOT "child*"[All Fields]

**Web of Science full search string (1643 results):**

TS=("Bariatric Surgery" OR "Obesity surgery" OR "Morbid obesity surgery") AND TS=("Gastric Bypass" OR "Gastric Bypass methods" OR "Gastric Bypass adverse effects" OR "One anastomosis gastric bypass" OR "OAGB" OR "Mini-gastric Bypass" OR "MGB" OR "omega loop gastric bypass" OR "olga" OR "Single anastomosis gastric bypass" OR "SAGB") AND TS=("clinical trial" OR "controlled clinical trial" OR "meta-analysis" OR "observational study" OR "systematic review") NOT TS=("pregnant*" OR "child*")

Refined by: Letter or Correction or Data Paper or Proceeding Paper or Editorial Material or Meeting Abstract (Exclude – Document Types)

**Embase full search string (1846 results):**

('bariatric surgery'/exp OR 'bariatric surgery' OR 'obesity'/exp/dm_su OR 'morbid obesity'/exp/mj/dm_su) AND ('gastric bypass surgery'/exp OR ('gastric bypass surgery'/exp/mj AND 'methodology'/de) OR 'gastric bypass surgery'/exp/mj/dd_ae OR 'one anastomosis gastric bypass' OR 'oagb' OR 'mini-gastric bypass' OR 'mgb' OR 'omega loop gastric bypass' OR 'olgb' OR 'single anastomosis gastric bypass' OR 'sagb') AND ('clinical trial':it OR 'controlled clinical trial':it OR 'meta analysis':it OR 'observational study':it OR 'systematic review') AND 'human'/exp NOT 'pregnant*' NOT 'child'/exp NOT 'child*' NOT ('letter':it OR 'editorial':it OR 'comment':it OR 'note':it OR 'news':it OR 'case reports':it OR 'conference abstract':it OR 'conference paper':it OR 'conference review':it OR 'chapter':it OR 'erratum':it OR 'tombstone':it OR 'short survey':it)

1. **Screening**

**ASReview**

To optimize the article selection process in this literature review, we used ASReview (version 2.0.2), an open-source machine learning framework designed to speed up and optimize the screening of titles and abstracts. It uses an Active learning algorithm, in which, based on the settings of the model, the algorithm can prioritize the most relevant records based on the user's decisions, thereby significantly reducing the number of records to be screened to find an estimated of at least 95% of the relevant records in the dataset. Unlike fully automated methods, ASReview integrates the interaction between the researcher and the algorithm to ensure efficient and transparent screening. (1)

**Prior knowledge**

For each section of our article, the model used was trained using a combination of title texts and abstracts of records extracted from an initial PubMed query (1663 records). **Supplementary** **Table 1** presents a selection of articles extracted from the Medline and PubMed Central databases as part of the initial model training process in ASReview. In this case, the titles and abstracts included in the table, which correspond to 10 relevant and 10 irrelevant articles, were previously selected by three reviewers. This process ensured that the articles complied with the topic and outcome indicated, constituting the necessary prior knowledge to optimize the active learning model in screening.

**Model**

The ELAS Ultra model with Term Frequency-Inverse Document Frequency (TF-IDF) feature extraction, Naive Bayes classifier, Maximum query strategy, and balanced balancer were used in this study.

**Stopping criterion**

It was decided to stop searching for relevant articles on ASReview once a threshold of 20 consecutive articles marked as irrelevant was reached. This stopping criterion was selected to prioritize reviewer time efficiency, due to the limited time resources available for the conduction of this review.

**Review update**

A review update was conducted on 28 May 2025 by repeating the PubMed query (1720 results) and adding the Embase (1846 results) and Web of Science (1643 results) searches. After deduplication (3791 records), all labels from the initial ASReview title and abstract screening were imported into the updated dataset which was assessed by screeners with ASReview.

**References**

1. Van de Schoot R, De Bruin J, Schram R, Zahedi P, De Boer J, Weijdema F, et al. An open source machine learning framework for efficient and transparent systematic reviews. Nat Mach Intell. February 1, 2021; 3(2):125-33.
