# Supplementary material for: One anastomosis gastric bypass (OAGB): a scoping review
Source: BMC Surg. 2025 Oct 10;25:471. doi: 10.1186/s12893-025-03215-x (PMC12512307; doi:10.1186/s12893-025-03215-x)
Supplement: Supplementary file 2 — Supplementary Material 2: Supplementary Tables 1 to 4. [file 12893_2025_3215_MOESM2_ESM.docx]

Supplementary Tables

Table of Contents

[**Supplementary Table 1** 2](#_Toc206434279)

[**Supplementary Table 2** 4](#_Toc206434280)

[**Supplementary Table 3** 10](#_Toc206434281)

[**Supplementary Table 4** 13](#_Toc206434282)

**Supplementary Table 1**. Digital object identifier (DOI) of initial records used as prior knowledge for ASReview training.

| **Prior knowledge for gastro-esophageal reflux disease section** | |
| --- | --- |
| **Prior knowledge relevant papers** | **Prior knowledge irrelevant papers** |
| 10.3390/nu16183210 | 10.24875/CIRU.23000545 |
| 10.1590/0102-6720202400021e1814 | 10.1007/s11695-024-07545-2 |
| 10.1007/s00464-024-10907-7 | 10.1007/s00464-024-11303-x |
| 10.1016/j.gassur.2024.01.038 | 10.1007/s11695-024-07526-5 |
| 10.3390/medicina60030405 | 10.1007/s11695-024-07424-w |
| 10.1007/s00464-024-10792-0 | 10.2337/dc24-0859 |
| 10.1007/s11695-024-07114-7 | 10.1007/s00464-024-11154-6 |
| 10.1016/S2213-8587(24)00035-4 | 10.1016/j.cpsurg.2024.101562 |
| 10.1016/S0140-6736(19)30475-1 | 10.1038/s41366-024-01585-5 |
| 10.1007/s11695-022-06002-2 | 10.1007/s11695-024-07465-1 |
| **Prior knowledge for complications section** | |
| **Prior knowledge relevant papers** | **Prior knowledge irrelevant papers** |
| 10.3390/nu16183210 | 10.24875/CIRU.23000545 |
| 10.1016/S2213-8587(24)00035-4 | 10.1007/s11695-024-07545-2 |
| 10.1016/S0140-6736(19)30475-1 | 10.1007/s00464-024-11303-x |
| 10.1007/s00464-024-10907-7 | 10.1007/s11695-024-07526-5 |
| 10.1016/j.gassur.2024.01.038 | 10.1007/s11695-024-07424-w |
| 10.1007/s11695-024-07114-7 | 10.2337/dc24-0859 |
| 10.1007/s11695-023-07050-y | 10.1007/s00464-024-11154-6 |
| 10.1007/s11695-024-07056-0 | 10.1016/j.cpsurg.2024.101562 |
| 10.1007/s00423-023-03175-x | 10.1038/s41366-024-01585-5 |
| 10.1002/oby.23852 | 10.1007/s11695-024-07465-1 |
| **Prior knowledge for remission of associated medical problems section** | |
| **Prior knowledge relevant papers** | **Prior knowledge irrelevant papers** |
| 10.1111/Sun.15974 | 10.1007/s11695-024-07465-1 |
| 10.3390/nu16183210 | 10.1186/s12893-024-02512-1 |
| 10.1590/0102-6720202400021e1814 | 10.1007/s11695-024-07382-3 |
| 10.1093/BJS/ZNAE219 | 10.3390/nu16142280 |
| 10.1007/s00464-024-10907-7 | 10.1007/S00104-024-02130-W |
| 10.1016/j.gassur.2024.01.038 | 10.1007/s00423-024-03413-w |
| 10.1016/S2213-8587(24)00035-4 | 10.1038/s41366-024-01591-7 |
| 10.1016/S0140-6736(19)30475-1 | 10.1016/j.jtha.2024.06.024 |
| 10.1007/s11695-022-06002-2 | 10.1016/j.soard.2024.05.010 |
| 10.1007/s11695-024-07114-7 | 10.1001/jamasurg.2024.2162 |
| **Prior knowledge for weight loss section** | |
| **Prior knowledge relevant papers** | **Prior knowledge irrelevant papers** |
| 10.1111/Sun.15974 | 10.1001/jamasurg.2022.2229 |
| 10.3390/nu16183210 | 10.1007/s11695-024-07545-2 |
| 10.1590/0102-6720202400021e1814 | 10.1007/s00464-024-11303-x |
| 10.1093/BJS/ZNAE219 | 10.1007/s11695-024-07526-5 |
| 10.1007/s00464-024-10907-7 | 10.1007/s11695-024-07424-w |
| 10.1016/j.gassur.2024.01.038 | 10.2337/dc24-0859 |
| 10.1016/S2213-8587(24)00035-4 | 10.1007/s00464-024-11154-6 |
| 10.1016/S0140-6736(19)30475-1 | 10.1016/j.cpsurg.2024.101562 |
| 10.1007/s11695-024-07114-7 | 10.1038/s41366-024-01585-5 |
| 10.3390/medicina60020256 | 10.1007/s11695-024-07465-1 |

**Supplementary Table 2**. Description of systematic reviews of OAGB evaluating at least one of the following: weight loss, remission of associated medical problems, complications, and GERD.

| **First Author and Publication Year** | **Study design** | **Outcome description** |
| --- | --- | --- |
| Mohammad Kermansaravi, 2025 [1] | Systematic Review and Meta-Analysis | Incidence results of iron deficiency anemia following OAGB with evaluation of the association of BLP as a variant in its occurrence. |
| Nikolaos Kapellas, 2025 [2] | Systematic Review and Meta-Analysis | Evaluation of the efficacy in controlling and resolving GERD symptoms in patients undergoing OAGB vs RYGB, as well as the rate of reflux-associated complications (esophagitis, Barrett's esophagus) and surgical reinterventions. |
| Yusuf Ahmed, 2025 [3] | Systematic Review and Meta-Analysis | Evaluation of OAGB and RYGB as primary procedures in patients with obesity, comparing outcomes in weight loss, resolution of associated medical problems, early and late complications, operative time, hospital stay, prevalence of reflux, and marginal ulcers. |
| Piotr Małczak, 2025 [4] | Systematic Review and Meta-Analysis | Effectiveness of different bariatric surgery modalities (SADI-S, OAGB, re-LSG), including percentage of excess weight loss, total weight loss percentage, and postoperative morbidity and mortality rates. |
| G Santoro,  2025 [5] | Systematic Review and Meta-Analysis | Comparison of outcomes between OAGB and RYGB as revisional surgery after failed SG, evaluating total weight loss, excess weight loss, operative time, diabetes mellitus remission, postoperative gastroesophageal reflux rate, and incidence of postoperative complications. |
| Xiaoyu Zang, 2025 [6] | Systematic Review and Meta-Analysis | Comparison of outcomes between OAGB and BPD in diabetes remission (complete/partial), weight loss, and safety profile. |
| Mohamed Abd Alla Salman, 2024 [7] | Systematic Review and Meta-Analysis | Comparison of SADI vs OAGB vs RYGB vs re-SG in weight loss, complications, and incidence of reflux. |
| Mohammad Kermansaravi, 2024 [8] | Systematic Review and Meta-Analysis | Comparison of the efficacy of OAGB vs SG and RYGB for remission and control of type 2 diabetes mellitus, as well as evaluation of adverse events and complications. |
| Tiago Rafael Onzi, 2024 [9] | Systematic Review and Meta-Analysis | Comparison of excess weight loss, changes in BMI, type 2 diabetes remission, complication rates, and GERD between procedures. Analysis of 1-year and 5-year outcomes in patients undergoing OAGB, RYGB, and OS. |
| Yusuf Ahmed, 2024 [10] | Systematic Review and Meta-Analysis | Comparison of the efficacy and safety of SADI vs OAGB in the context of revisional surgery after failed SG, evaluating weight loss after revisional surgery, improvement in associated medical problems, nutritional deficiencies, and postoperative complication rates. |
| Amir Hossein, 2023 [11] | Systematic Review and Meta-Analysis | Incidence of GERD in patients undergoing OAGB, the need for treatment for GERD in patients, endoscopic findings, and the time interval between the onset of GERD and the performance of OAGB surgery. |
| Zujun Ding, 2023 [12] | Systematic Review and Meta-Analysis | Remission of type 2 diabetes, improvement in hypertension and fasting plasma glucose levels, percentage loss of excess BMI, percentage of excess weight loss, percentage of total weight loss, changes in body mass index, and comparison of efficacy and safety between OAGB and OS. |
| Ali Esparham, 2023 [13] | Systematic Review and Meta-Analysis | Incidence of gastroesophageal reflux after OAGB, changes in preoperative reflux status, rates of esophagitis and Barrett's esophagus, and surgical revisions due to reflux, gastritis, and marginal ulcer. Comparison of reflux rates between OAGB, RYGB and SG. |
| Barzin Maryam, 2023 [14] | Systematic Review and Meta-Analysis | Comparison of the percentage of excess weight loss and percentage of total weight loss between OAGB and OS in patients with BMI greater than 50. Evaluation of differences in operative time, hospital stay, and complications between OAGB and OS. Remission rates of type 2 diabetes and hypertension between OAGB and OS. Short- and medium-term efficacy of OAGB compared to OS in patients with severe clinical obesity. |
| Shiela Lee, 2023 [15] | Systematic Review and Meta-Analysis | Incidence of marginal ulcers after OAGB. Evaluation of risk factors, history of GERD, and use of proton pump inhibitors as postoperative prophylaxis. |
| Mohamed AbdAlla Salman, 2023 [16] | Systematic Review and Meta-Analysis | Comparison of OAGB outcomes with 150 cm versus 200 cm GLP in terms of weight loss, remission of associated medical problems, and adverse nutritional effects. |
| Muhammad Ali, 2023 [17] | Systematic Review and Meta-Analysis | Comparison of outcomes between OAGB and OS, including excess weight loss at 1, 2, 3, 4, and 5 years, remission rates of dyslipidemia, hypertension, type 2 diabetes, and bleeding, as well as the incidence of GERD and leakage in patients undergoing MBS |
| Hatem K El-Gohary, 2023 [18] | Systematic Review and Meta-Analysis | Comparison of postoperative outcomes of conversions from failed restrictive procedures to RYGB, OAGB, or re-SG, in terms of efficacy (weight loss) and safety (common complications). |
| Maurizio De Luca, 2023 [19] | Systematic Review and Meta-Analysis | Evaluation and comparison of weight loss effectiveness, metabolic health improvement, and adverse effects in patients undergoing different bariatric surgeries. (RYGB, BPD-DS, OAGB, SG). |
| Erfana Tasdighi, 2022 [20] | Systematic Review and Meta-Analysis | Evaluation of the effect of BPL on weight loss, postoperative complications, and remission of associated medical problems in patients undergoing OAGB. |
| Xianting Li,  2022 [21] | Systematic Review and Meta-Analysis | Comparison of the efficacy and safety of OAGB vs RYGB in percentage loss of excess weight loss and changes in BMI and remission type 2 diabetes and dyslipidemia. Evaluation of early and late postoperative complications, operative time between OAGB and RYGB. |
| Yichen Li, 2022 [22] | Systematic Review and Meta-Analysis | Evaluation of the efficacy of GLP length in OAGB, including excess weight loss, total weight loss, incidence of vitamin D deficiency, hypoalbuminemia, and short- and long-term changes in BMI (BMI). |
| Chao Xing Lin, 2022 [23] | Systematic Review | Outcomes in type 2 diabetes mellitus remission, changes in BMI, lipid levels, and blood pressure, comparing metabolic surgery with restrictive procedures, medical interventions, and lifestyle modifications. |
| Claudia Cosentino, 2021 [24] | Systematic Review and Meta-Analysis | Body weight reduction at 1 year, BMI decrease, adverse events, and surgical safety in patients undergoing different bariatric interventions (SG, RYGB, LAGB). |
| Piotr Małczak, 2021 [25] | Systematic Review and Meta-Analysis | Comparison of overall quality of life at 1, 2, 3, and 5 years following bariatric surgery versus non-surgical interventions, and among different surgical procedures (SG, RYGB, OAGB). |
| Barbara Cresci, 2020 [26] | Systematic Review and Meta-Analysis | Comparison of outcomes from different metabolic surgeries in patients with diabetes and overweight/obesity, using changes in HbA1c, FPG, and complete or partial remission of type 2 diabetes mellitus as parameters. |
| Maria Neves Carmona, 2021 [27] | Systematic Review and Meta-Analysis | Outcomes in weight loss and type 2 diabetes mellitus remission comparing RYGB, SG, BPD-DS, and medical treatment. |
| Li Ding, 2020 [28] | Systematic Review and Meta-Analysis | Remission of type 2 diabetes mellitus based on outcomes related to changes in body weight, blood pressure, lipid profile, and overall metabolic control in patients who underwent bariatric procedures (RYGB, BPD, LSG, LAGB). |
| Dimitrios E. Magouliotis, 2019 [29] | Systematic Review and Meta-Analysis | Comparison of clinical outcomes between OAGB and RYGB such as operative time, leakage, marginal ulcers, dumping, bowel obstruction, check-ups, excess weight loss, and mortality in patients with clinical obesity. |
| Desheng Jia, 2019 [30] | Systematic Review and Meta-Analysis | Comparison of clinical outcomes between OAGB and RYGB, including loss of excess BMI at 2 years, remission of type 2 diabetes, adverse events, and the impact of biliopancreatic loop length in patients with clinical obesity. |
| Chan Hyuk Park, 2019 [31] | Systematic Review and Meta-Analysis | Comparison of different bariatric procedures (RYGB, OAGB, BPD/DS, etc.) with outcomes in type 2 diabetes mellitus remission, weight loss, and safety profile. |
| S Kodama,  2018 [32] | Systematic Review and Meta-Analysis | Outcomes in the remission rate of type 2 diabetes mellitus following bariatric procedures (BPD, BPD-DS, RYGB, OAGB, SG, adjustable gastric banding, greater curvature plication). |
| Dimitrios E Magouliotis, 2017 [33] | Systematic Review and Meta-Analysis | Comparison of the effectiveness of OAGB versus SG in weight loss and resolution of metabolic diseases, along with the postoperative complication profile. |
| Mohammad Kermansaravi, 2025 [34] | Systematic Review | Incidence of dumping syndrome following OAGB surgery, the stages of dumping, associated clinical presentation, and the various treatment modalities. |
| G Balamurugan, 2023 [35] | Systematic Review | Total weight loss and excess weight loss, resolution of associated medical problems and complication rates among three bariatric procedures: RYGB, OAGB and SADI-S. |
| Rachel Xue Ning Lee, 2022 [36] | Systematic Review | Evaluation of revisional surgery after OAGB by GERD, including frequency of revisions, techniques used such as Roux-en-Y conversion, Braun anastomosis, and fundoplication of the excluded stomach, and resolution of reflux symptoms |
| Shahbakht, Ilyas 2020 [37] | Systematic Review | Comparison of bariatric surgeries versus medical and lifestyle interventions, as well as among different types of surgical procedures, evaluating outcomes in terms of type 2 diabetes mellitus remission. |
| Chetan D. Parmar, 2019 [38] | Systematic Review | Evaluation of the safety and efficacy of OAGB in patients with obesity (BMI > 50 kg/m²). |
| Chetan D. Parmar, 2018 [39] | Systematic review | Evaluation of cumulative outcomes of 12,807 OAGB procedures in patients with obesity. Mortality rates, leakage, marginal ulcers, anemia, postoperative gastroesophageal reflux, and malnutrition. Loss of excess weight at 6, 12, 24 and 60 months. Resolution of type 2 diabetes and hypertension. |

**GERD:** Gastroesophageal Reflux Disease; **OAGB:** One Anastomosis Gastric Bypass; **RYGB:** Roux-en-Y Gastric Bypass; **SADI-S**: Single Anastomosis Duodeno-Ileal Bypass with Sleeve Gastrectomy; **SG:** Sleeve Gastrectomy; **BMI**: Body Mass Index; **BPL**: Biliopancreatic Loop; **TSBL**: Total Length of the Small Intestine; **BAROS:** Bariatric Analysis and Reporting Outcome System; **BPD:** Biliopancreatic diversion; **BPD-DS:** Biliopancreatic diversion with duodenal switch; **LAGB:** Laparoscopic adjustable gastric banding; **HbA1c:** Glycated hemoglobin; **FPG:** Fasting plasma glucose; **re-LSG**: Re-Sleeve gastrectomy.

**Supplementary Table 3**. Characteristics of the key randomized control trials of OAGB and those not priorly included in other systematic literature reviews with meta-analyses of weight loss, remission of associated medical problems, complications, or GERD.

| **First Author and Publication Year** | **Outcome Description** |
| --- | --- |
| Oral Ospanov (2024) [40] | Comparison of bariatric and antireflux efficacy of OAGB with and without fundoplication using the excluded stomach (FundoRingOAGB vs NissenOAGB vs OAGB). Comparison of BMI reduction, GERD symptoms (GERD-HRQL), and VISICK score at 24 months. Evaluation of the effectiveness of different antireflux techniques in improving heartburn and overall GERD symptom scores in patients with obesity undergoing OAGB. |
| Mohammad Kermansaravi (2024) [41] | Comparison of the efficacy of OAGB vs RYGB in patients with clinical obesity and mild to moderate GERD. Comparison of operative time, complications, and weight loss between OAGB and RYGB. Effects of OAGB and RYGB on reducing GERD symptoms, assessed by questionnaires, upper endoscopy, 24-hour pH monitoring, and manometry. |
| Nienke Slagter (2024) [42] | Evaluation of BPL personalization based on TSBL length compared to a fixed length of 150 cm in patients undergoing OAGB. Comparison of 1-year percentage total weight loss between groups with standard GLP 150 cm and customized GLP 150 cm vs 180 cm vs 210 cm according to TSBL. |
| Maud Robert (2024) [43] | Comparison of percentage loss of excess BMI and 5-year metabolic and safety outcomes between OAGB and RYGB. Adverse event analysis, with particular attention to the incidence of GERD after OAGB and RYGB. |
| Tarik Delko  (2024) [44] | Comparison of 12-month excess weight loss between OAGB and RYGB. Evaluation of safety, rates of marginal ulcers, and late complications between OAGB and RYGB. Remission of associated medical problems, with greater remission of GERD in RYGB and better glycemic control in OAGB. |
| Mayank Jain  (2024) [45] | Comparison of the 7-year results between the LSG and the OAGB using the BAROS system (Bariatric Analysis and Reporting Outcome System). Assessment of total body weight loss, BMI, percentage of excess weight loss, and percentage of total weight loss. |
| Mohamed AbdAlla S.  (2023) [46] | Comparison of weight loss and remission of type 2 diabetes, hypertension, obstructive sleep apnea, joint and low back pain between OAGB and RYGB with BPL. Evaluation of reflux symptoms in patients undergoing OAGB and their management with proton pump inhibitors. |
| A van Rijswijk  (2022) [47] | Comparison of the efficacy of OAGB vs RYGB in patients with obesity and type 2 diabetes mellitus (T2DM). Comparison of glycemic control at 12 months, weight loss, surgical complications, quality of life, and gastrointestinal symptoms between OAGB and RYGB. Assessment of T2DM remission mechanisms through metabolic, microbial, and immunological markers, including repeated blood tests, mixed meal tolerance tests, liver biopsy (for NAFLD/NASH), microbiome profiling, bile acid composition, cardiovascular performance, and gallstone formation. |
| Moheb S. Eskandaros  (2021) [48] | Comparison of the efficacy of OAGB vs RYGB in patients with clinical obesity and mild to moderate GERD. Comparison of operative time, complications, and weight loss between OAGB and RYGB. Effects of OAGB and RYGB on reducing GERD symptoms, assessed by questionnaires, upper endoscopy, 24-hour pH monitoring, and manometry. |
| Luis Level  (2020) [49] | Comparison of outcomes between OAGB and RYGB, including excess weight loss at 1, 6, 12 months, and 5 years, remission of type 2 diabetes, insulin resistance, dyslipidemia, and GERD, operative time, use of surgical staples, postoperative pain, and hospital stay in patients undergoing bariatric surgery. |
| Maud Robert  (2019) [50] | Comparison of the efficacy and safety of OAGB vs RYGB in percentage loss of excess BMI and metabolic improvement at 2 years. |
| Tuure Saarinen et al.  (2019) [51] | Evaluation of postoperative bile reflux after OAGB surgery, trial without control group. |

**OAGB:** One Anastomosis Gastric Bypass; **RYGB:** Roux-en-Y Gastric Bypass; **BMI**: Body Mass Index; **GERD:** Gastroesophageal Reflux Disease; **GERD-HRQL**: Gastroesophageal Reflux Disease – Health-Related Quality of Life; **VISICK**: Visick score; **BPL**: Biliopancreatic Limb; **TSBL**: Total Small Bowel Length; **GLP**: Gastrojejunal Limb Personalization; **LSG**: Laparoscopic Sleeve Gastrectomy; **BAROS:** Bariatric Analysis and Reporting Outcome System; **T2DM**: Type 2 Diabetes Mellitus; **NAFLD**: Non-Alcoholic Fatty Liver Disease; **NASH**: Non-Alcoholic Steatohepatitis; **PPIs**: Proton Pump Inhibitors

**Supplementary Table 4**. Description of observational studies of OAGB evaluating at least one of the following: weight loss, remission of associated medical problems, complications, and GERD.

| **First author and publication year** | **Study Design** | **Outcome Description** |
| --- | --- | --- |
| Stefania Gorini, 2025 [52] | Retrospective Observational Study | Evaluation of long-term weight loss efficacy, nutritional deficiencies, and patient-reported satisfaction in patients undergoing LSG, OAGB, and RYGB. Assessment of excess weight loss (%EWL) at 60 months, prevalence of iron and vitamin D deficiencies, impact of surgical revisions (e.g., LSG to RYGB or MGB) on outcomes, and patient willingness to repeat surgery. |
| Sara Sadeghi, 2025 [53] | Cohort Study | Evaluation of the rates and predictors of resolution and relapse of metabolic dysfunction-associated steatotic liver disease (MASLD) in patients undergoing sleeve gastrectomy (SG) or one-anastomosis gastric bypass (OAGB), including hepatic steatosis percentage, 12-month excess weight loss (EWL%), preoperative fat mass percentage (FM%), and patient age. Resolution and relapse rates were assessed per 1000 person-months over a median follow-up of 2.2 years. |
| Sneha Makkapati,  2024 [54] | Retrospective Observational Study | Evaluation of long-term efficacy, safety, nutritional status, and resolution of obesity-related comorbidities in patients undergoing one-anastomosis gastric bypass (OAGB). Assessment included 10-year excess weight loss (EWL), body mass index (BMI) reduction, resolution rates of type 2 diabetes mellitus (DM), hypertension (HTN), and obstructive sleep apnea (OSA), and incidence of severe malnutrition. |
| Hsin-Mei Pan, 2024 [55] | Retrospective Observational Study | Evaluation of the long-term efficacy, safety, and cardiovascular risk reduction following one-anastomosis gastric bypass (OAGB) and single-anastomosis duodenal-jejunal bypass with sleeve gastrectomy (SADJB-SG). Outcomes included 3-year weight loss, diabetes remission, improvements in hypertension and dyslipidemia, and changes in 10-year and lifetime risks of major adverse cardiovascular events (MACE) assessed using the Taiwan MACE risk prediction model and the China-PAR project model. |
| Sara Sadeghi, 2024 [56] | Prospective Observational Study | Evaluation of the rates and predictors of remission and relapse of type 2 diabetes in patients undergoing OS or OAGB, including glycated hemoglobin levels, fasting plasma glucose, use of hypoglycemic agents/insulin, and time to remission or relapse. |
| Guillermo Borjas, 2022 [57] | Prospective Observational Study | Evaluation of the safety and feasibility of magnetic surgical system assistance during various steps of single-port and reduced-port bariatric procedures, including sleeve gastrectomy (SG), one-anastomosis gastric bypass (OAGB), Roux-en-Y gastric bypass (RYGB), and revisional surgeries. Outcomes assessed included surgical time, intraoperative complications, and 30-day postoperative morbidity and mortality. |
| Fatemeh Bourbour, 2021 [58] | Cohort Study | Evaluation of serum vitamin D trends from baseline to 12 months after one-anastomosis gastric bypass (OAGB) in patients receiving >800 IU/day of supplementation. Outcomes included changes in vitamin D levels and associations with anthropometric, biochemical, and metabolic parameters including body composition, lipid profile, glycemic indices, parathormone, and basal metabolic rate (BMR). |
| Nienke Slagter, 2021 [59] | Cohort Study | Evaluation of the impact of an anti-reflux suture on the incidence of postoperative reflux and need for conversion to Roux-en-Y gastric bypass (RYGB) in patients undergoing one-anastomosis gastric bypass (OAGB). Outcomes included rates of reflux symptoms, conversion surgery for reflux, and the influence of preoperative gastroesophageal reflux disease (GERD) as a predictive factor. |
| Jose-Maria Jimenez (2020) [60] | Cross-sectional Observational study | Evaluation of changes in weight and body composition during the first postoperative year in patients undergoing one-anastomosis gastric bypass (OAGB). Parameters assessed included body weight, body mass index (BMI), fat mass, fat-free mass, bone mass, and total body water. Outcomes aimed to support improved postoperative monitoring and long-term follow-up strategies. |
| Artur Marc-Hernández (2020) [61] | Prospective Observational Study | Evaluation of changes in body composition and bone mass in patients undergoing one-anastomosis gastric bypass (OAGB), using bioelectrical impedance analysis at baseline, 6 months, and 12 months postoperatively. Outcomes included excess BMI loss, fat-free mass (FFM) reduction, and bone mass decrease, with a focus on the magnitude and clinical relevance of these changes over time. |
| Jaime Ruiz-Tovar, 2020 [62] | Prospective Observational Study | Evaluation of the influence of biliopancreatic limb (BPL) and common limb (CL) lengths, and their ratios to total bowel length (TBL), on weight loss and nutritional deficits in patients undergoing one-anastomosis gastric bypass (OAGB). Outcomes included units of BMI lost (UBMIL), achievement of ideal BMI (≤ 25 kg/m²) at 1, 2, and 5 years, remission of comorbidities, and incidence of protein or calorie malnutrition. |
| Salvador Navarrete, 2019 [63] | Prospective Observational Study | Evaluation of weight loss and metabolic improvements, including fasting glycemia and HbA1c levels, in mild obese patients with type 2 diabetes mellitus or insulin resistance undergoing one-anastomosis gastric bypass (OAGB/MGB). Outcomes assessed at 12 months included excess weight loss percentage and diabetes remission rates. |
| Sonja Chiappetta, 2018 [64] | Retrospective Observational Study | Evaluation of inflammatory marker changes, including C-reactive protein (CRP) and leukocyte count, after sleeve gastrectomy (SG), Roux-en-Y gastric bypass (RYGB), and one-anastomosis gastric bypass (OAGB). Outcomes assessed at 6 months postoperatively included total body weight loss (TBWL%) and changes in CRP and leukocyte levels to compare the impact of different bariatric procedures on chronic inflammation. |
| Miguel A. Carbajo, 2017 [65] | Cohort Study | Evaluation of the 6-12 year results of the OAGB technique, including operative time, intraoperative and postoperative complications, excess weight loss, changes in BMI, remission or improvement of comorbidities, quality of life, and readmission and mortality rates. |
| Miguel Carbajo, 2005 [66] | Cohort Study | Evaluation of OAGB outcomes in the first 209 patients, including operative time, intraoperative and postoperative complications such as conversions, reoperations, anastomotic leakage, acute pancreatitis and iron deficiency anemia, excess weight loss at 1 and 2 years, and mortality rates. |
| Robert Rutledge, 2001 [67] | Cohort Study | Evaluation of MGB outcomes, including excess weight loss at 6 months, 1 year, and 2 years, operative time, hospital stay, complication rates such as deep vein thrombosis, pulmonary embolism, and leakage, in-hospital mortality, and improvement or resolution of associated medical diseases. |

**LSG**: Laparoscopic Sleeve Gastrectomy.; **OAGB**: One-Anastomosis Gastric Bypass.; **RYGB**: Roux-en-Y Gastric Bypass.; **MGB**: Mini Gastric Bypass.; **MASLD**: Metabolic dysfunction-associated steatotic liver disease.; **EWL%**: Excess Weight Loss percentage.; **FM%**: Fat Mass percentage.; **DM**: Diabetes Mellitus.; **HTN**: Hypertension.; **OSA**: Obstructive Sleep Apnea.; **BMI**: Body Mass Index.; **MACE**: Major Adverse Cardiovascular Events.; **SADJB-SG**: Single-Anastomosis Duodenal-Jejunal Bypass with Sleeve Gastrectomy.; **TBWL%**: Total Body Weight Loss percentage.; **CRP**: C-Reactive Protein.; **HbA1c**: Glycated Hemoglobin.; **FM**: Fat Mass.; **FFM**: Fat-Free Mass.; **BMR**: Basal Metabolic Rate.; **GERD**: Gastroesophageal Reflux Disease.; **UBMIL**: Units of BMI Lost.; **BPL**: Biliopancreatic Limb.; **CL**: Common Limb.; **TBL**: Total Bowel Length.; **RPI**: Peripheral Insulin Resistance.; **SG**: Sleeve Gastrectomy.; **SPSG**: Single-Port Sleeve Gastrectomy.

**References**

1. Kermansaravi M, Shahsavan M, Hage K, Taskin HE, ShahabiShahmiri S, Poghosyan T, et al. Iron deficiency anemia after one anastomosis gastric bypass: A systematic review and meta-analysis. Surg Endosc. 2025;39:1509–22. https://doi.org/10.1007/s00464-025-11535-5.

2. Kapellas N, Alkhalil S, Senkal M. Efficacy of One-Anastomosis Gastric Bypass Versus Roux-en-Y Gastric Bypass for Gastroesophageal Reflux Disease: A Systematic Review and Meta-analysis of Randomized Controlled Trials. Obes Surg. 2024;34:4563–72. https://doi.org/10.1007/s11695-024-07571-0.

3. Ahmed Y, Ataya K, Almubarak A, Almubarak I, Ali M, Yusuf W, et al. One Anastomosis Gastric Bypass Versus Roux‑en‑Y Gastric Bypass for Obesity: An Updated Meta‑analysis and Systematic Review of Randomized Controlled Trials. Obes Surg. 2025;35:1438–46. https://doi.org/10.1007/s11695-025-07776-x.

4. Małczak P, Shim SR, Wysocki M, Rymarowicz J, Wierdak M, Pędziwiatr M, et al. Comparison of different revisional surgeries after sleeve gastrectomy: A network meta-analysis. Obes Rev. 2025;26:e13930. https://doi.org/10.1111/obr.13930.

5. Santoro G, Alfred J, Rehman A, Sheriff N, Naing H, Tandon A. Revisional bariatric surgery following sleeve gastrectomy: a meta-analysis comparing Roux-en-Y gastric bypass and one anastomosis gastric bypass. Ann R Coll Surg Engl. 2025;107:180–7. https://doi.org/10.1308/rcsann.2024.0054.

6. Zang X, Lin T, Ma J, Zhang Y, Zhang B, Huang Y, et al. Comparison of Benefits and Risks of Metabolic Surgery for Long-Term (5 Years) Weight Loss and Diabetes Remission in Overweight/Obese Patients With Type 2 Diabetes: A Systematic Review and Network Meta-Analysis of Randomized Trials. Diabetes Metab Res Rev. 2025;41:e70033. https://doi.org/10.1002/dmrr.70033.

7. Salman MA, Salman A, Elewa A, Elsherbiny M, Tourkey M, Chikukuza S, et al. Outcomes of revisional surgery options after inadequate sleeve gastrectomy: A comprehensive network meta-analysis. World J Surg. 2024;48:2040–57. https://doi.org/10.1002/wjs.12293.

8. Kermansaravi M, Chiappetta S, Kassir R, Bosco A, Giudicelli X, Lainas P, et al. Efficacy of One Anastomosis Gastric Bypass Versus Sleeve Gastrectomy and Roux-en-Y Gastric Bypass for the Treatment of Type 2 Diabetes Mellitus: a Systematic Review and Meta-Analysis of Randomized Clinical Trials. Obes Surg. 2024;34:4555–62. https://doi.org/10.1007/s11695-024-07564-z.

9. Onzi TR, Salgado Júnior W, Bastos EL de S, Dantas ACB, Silva LB, Oliveira Neto AA de, et al. EFFICACY AND SAFETY OF ONE ANASTOMOSIS GASTRIC BYPASS IN SURGICAL TREATMENT OF OBESITY: SYSTEMATIC REVIEW AND META-ANALYSIS OF RANDOMIZED CONTROLLED TRIALS. Arq Bras Cir Dig. 2024;37:e1814. https://doi.org/10.1590/0102-6720202400021e1814.

10. Ahmed Y, Ataya K, Almubarak I, Ali M, Almubarak A, Yusuf W, et al. Laparoscopic Single Anastomosis Duodeno-Ileal Bypass Versus One Anastomosis Gastric Bypass as Revisional Procedures after Sleeve Gastrectomy: Meta-analysis and Systematic Review. Obes Surg. 2024;34:4405–12. https://doi.org/10.1007/s11695-024-07584-9.

11. Davarpanah Jazi AH, Shahabi S, Sheikhbahaei E, Tolone S, Skalli ME, Kabir A, et al. A systematic review and meta-analysis on GERD after OAGB: rate, treatments, and success. Expert Rev Gastroenterol Hepatol. 2023;17:1321–32. https://doi.org/10.1080/17474124.2023.2296992.

12. Ding Z, Jin L, Song Y, Feng C, Shen P, Li H. Comparison of single-anastomosis gastric bypass and sleeve gastrectomy on type 2 diabetes mellitus remission for obese patients: A meta-analysis of randomized controlled trials. Asian J Surg. 2023;46:4152–60. https://doi.org/10.1016/j.asjsur.2023.03.062.

13. Esparham A, Ahmadyar S, Zandbaf T, Dalili A, Rezapanah A, Rutledge R, et al. Does One-Anastomosis Gastric Bypass Expose Patients to Gastroesophageal Reflux: a Systematic Review and Meta-analysis. Obes Surg. 2023;33:4080–102. https://doi.org/10.1007/s11695-023-06866-y.

14. Barzin M, Ebadinejad A, Aminian A, Khalaj A, Ghazy F, Koohi F, et al. Does one-anastomosis gastric bypass provide better outcomes than sleeve gastrectomy in patients with BMI greater than 50? A systematic review and meta-analysis. Int J Surg. 2023;109:277–86. https://doi.org/10.1097/JS9.0000000000000203.

15. Lee S, Supparamaniam S, Varghese C, Mahawar K. Marginal Ulcers Following One-Anastomosis Gastric Bypass: a Systematic Review and Meta-analysis. Obes Surg. 2023;33:2884–97. https://doi.org/10.1007/s11695-023-06762-5.

16. Salman MA, Salman A, Assal MM, Elsherbiney M, Tourky M, Elewa A, et al. One Anastomosis Gastric Bypass (OAGB) with a 150-cm Biliopancreatic Limb (BPL) Versus a 200-cm BPL, a Systematic Review and Meta-analysis. Obes Surg. 2023;33:1846–56. https://doi.org/10.1007/s11695-023-06556-9.

17. Ali M, Wang Y, Ji J, Wang W, Wang D. One Anastomosis Gastric Bypass Versus Sleeve Gastrectomy for Obesity: a Systemic Review and Meta-analysis. J Gastrointest Surg. 2023;27:2226–44. https://doi.org/10.1007/s11605-023-05782-x.

18. El-Gohary HK, Abdelbaeth A, Sayed HA, Kamal A. Revisional surgeries after failed restrictive bariatric operations: a meta-analysis. The Egyptian Journal of Surgery. 2023;42:302. https://doi.org/10.4103/ejs.ejs_63_23.

19. De Luca M, Zese M, Silverii GA, Ragghianti B, Bandini G, Forestieri P, et al. Bariatric Surgery for Patients with Overweight/Obesity. A Comprehensive Grading Methodology and Network Metanalysis of Randomized Controlled Trials on Weight Loss Outcomes and Adverse Events. Obes Surg. 2023;33:4147–58. https://doi.org/10.1007/s11695-023-06909-4.

20. Tasdighi E, Barzin M, Mahawar KK, Hosseinpanah F, Ebadinejad A, Taraghikhah N, et al. Effect of Biliopancreatic Limb Length on Weight Loss, Postoperative Complications, and Remission of Comorbidities in One Anastomosis Gastric Bypass: a Systematic Review and Meta-analysis. Obes Surg. 2022;32:892–903. https://doi.org/10.1007/s11695-021-05848-2.

21. Li X, Hu X, Fu C, Han L, Xie M, Ouyang S. Efficacy and Safety of One Anastomosis Gastric Bypass Versus Roux-en-Y Gastric Bypass for Obesity: a Meta-analysis and Systematic Review. Obes Surg. 2023;33:611–22. https://doi.org/10.1007/s11695-022-06401-5.

22. Li Y, Gu Y, Jin Y, Mao Z. What Is the Efficacy of Short Length of Biliopancreatic Limb in One-Anastomosis Gastric Bypass? A Systematic Review and Meta-analysis of Short-Term Results. Obes Surg. 2022;32:1–9. https://doi.org/10.1007/s11695-022-06048-2.

23. Lin C, Yeong TJJ-M, Lim WH, Ng CH, Yau CE, Chin YH, et al. Comparison of mechanistic pathways of bariatric surgery in patients with diabetes mellitus: A Bayesian network meta-analysis. Obesity (Silver Spring). 2022;30:1380–90. https://doi.org/10.1002/oby.23453.

24. Cosentino C, Marchetti C, Monami M, Mannucci E, Cresci B. Efficacy and effects of bariatric surgery in the treatment of obesity: Network meta-analysis of randomized controlled trials. Nutr Metab Cardiovasc Dis. 2021;31:2815–24. https://doi.org/10.1016/j.numecd.2021.06.018.

25. Małczak P, Mizera M, Lee Y, Pisarska-Adamczyk M, Wysocki M, Bała MM, et al. Quality of Life After Bariatric Surgery-a Systematic Review with Bayesian Network Meta-analysis. Obes Surg. 2021;31:5213–23. https://doi.org/10.1007/s11695-021-05687-1.

26. Cresci B, Cosentino C, Monami M, Mannucci E. Metabolic surgery for the treatment of type 2 diabetes: A network meta-analysis of randomized controlled trials. Diabetes Obes Metab. 2020;22:1378–87. https://doi.org/10.1111/dom.14045.

27. Carmona MN, Santos-Sousa H, Lindeza L, Sousa-Pinto B, Nogueiro J, Pereira A, et al. Comparative Effectiveness of Bariatric Surgeries in Patients with Type 2 Diabetes Mellitus and BMI ≥ 25 kg/m2: a Systematic Review and Network Meta-Analysis. Obes Surg. 2021;31:5312–21. https://doi.org/10.1007/s11695-021-05725-y.

28. Ding L, Fan Y, Li H, Zhang Y, Qi D, Tang S, et al. Comparative effectiveness of bariatric surgeries in patients with obesity and type 2 diabetes mellitus: A network meta-analysis of randomized controlled trials. Obes Rev. 2020;21:e13030. https://doi.org/10.1111/obr.13030.

29. Magouliotis DE, Tasiopoulou VS, Tzovaras G. One Anastomosis Gastric Bypass Versus Roux-en-Y Gastric Bypass for Morbid Obesity: an Updated Meta-Analysis. Obes Surg. 2019;29:2721–30. https://doi.org/10.1007/s11695-019-04005-0.

30. Jia D, Tan H, Faramand A, Fang F. One Anastomosis Gastric Bypass Versus Roux-en-Y Gastric Bypass for Obesity: a Systematic Review and Meta-Analysis of Randomized Clinical Trials. Obes Surg. 2020;30:1211–8. https://doi.org/10.1007/s11695-019-04288-3.

31. Park CH, Nam S-J, Choi HS, Kim KO, Kim DH, Kim J-W, et al. Comparative Efficacy of Bariatric Surgery in the Treatment of Morbid Obesity and Diabetes Mellitus: a Systematic Review and Network Meta-Analysis. Obes Surg. 2019;29:2180–90. https://doi.org/10.1007/s11695-019-03831-6.

32. Kodama S, Fujihara K, Horikawa C, Harada M, Ishiguro H, Kaneko M, et al. Network meta-analysis of the relative efficacy of bariatric surgeries for diabetes remission. Obes Rev. 2018;19:1621–9. https://doi.org/10.1111/obr.12751.

33. Magouliotis DE, Tasiopoulou VS, Svokos AA, Svokos KA, Sioka E, Zacharoulis D. One-Anastomosis Gastric Bypass Versus Sleeve Gastrectomy for Morbid Obesity: a Systematic Review and Meta-analysis. Obes Surg. 2017;27:2479–87. https://doi.org/10.1007/s11695-017-2807-2.

34. Kermansaravi M, Shahsavan M, Amr B, Stier C, Parmar C, Chiappetta S. Dumping Syndrome After One Anastomosis Gastric Bypass-A Systematic Review. Obes Surg. 2025;35:2310–20. https://doi.org/10.1007/s11695-025-07860-2.

35. Balamurugan G, Leo SJ, Sivagnanam ST, Balaji Prasad S, Ravindra C, Rengan V, et al. Comparison of Efficacy and Safety Between Roux-en-Y Gastric Bypass (RYGB) vs One Anastomosis Gastric Bypass (OAGB) vs Single Anastomosis Duodeno-ileal Bypass with Sleeve Gastrectomy (SADI-S): a Systematic Review of Bariatric and Metabolic Surgery. Obes Surg. 2023;33:2194–209. https://doi.org/10.1007/s11695-023-06602-6.

36. Lee RXN, Rizkallah N, Chiappetta S, Stier C, Pouwels S, Sakran N, et al. Surgical Management of Gastro-oesophageal Reflux Disease After One Anastomosis Gastric Bypass - a Systematic Review. Obes Surg. 2022;32:4057–65. https://doi.org/10.1007/s11695-022-06301-8.

37. Ilyas S, Al-Refai R, Maharjan R, Diaz Bustamante L, Ghattas KN, Khan S. Bariatric Surgery and Type 2 Diabetes Mellitus: Assessing Factors Leading to Remission. A Systematic Review. Cureus. 2020;12:e9973. https://doi.org/10.7759/cureus.9973.

38. Parmar CD, Bryant C, Luque-de-Leon E, Peraglie C, Prasad A, Rheinwalt K, et al. One Anastomosis Gastric Bypass in Morbidly Obese Patients with BMI ≥ 50 kg/m2: a Systematic Review Comparing It with Roux-En-Y Gastric Bypass and Sleeve Gastrectomy. Obes Surg. 2019;29:3039–46. https://doi.org/10.1007/s11695-019-04034-9.

39. Parmar CD, Mahawar KK. One Anastomosis (Mini) Gastric Bypass Is Now an Established Bariatric Procedure: a Systematic Review of 12,807 Patients. Obes Surg. 2018;28:2956–67. https://doi.org/10.1007/s11695-018-3382-x.

40. Ospanov O, Zharov N, Yelembayev B, Duysenov G, Volchkova I, Sultanov K, et al. A Three-Arm Randomized Controlled Trial of Primary One-Anastomosis Gastric Bypass: With FundoRing or Nissen Fundoplications vs. without Fundoplication for the Treatment of Obesity and Gastroesophageal Reflux Disease. Medicina. 2024;60:405. https://doi.org/10.3390/medicina60030405.

41. Kermansaravi M, Shahsavan M, Ebrahimi R, Mousavimaleki A, Gholizadeh B, Valizadeh R, et al. Effect of anti-reflux suture on gastroesophageal reflux symptoms after one anastomosis gastric bypass: a randomized controlled trial. Surg Endosc. 2024;38:2562–70. https://doi.org/10.1007/s00464-024-10792-0.

42. Slagter N, van der Laan L, de Heide LJM, Jutte EH, Kaijser MA, Damen SL, et al. Effect of tailoring biliopancreatic limb length based on total small bowel length versus standard limb length in one anastomosis gastric bypass: 1-year outcomes of the TAILOR randomized clinical superiority trial. Br J Surg. 2024;111:znae219. https://doi.org/10.1093/bjs/znae219.

43. Robert M, Poghosyan T, Maucort-Boulch D, Filippello A, Caiazzo R, Sterkers A, et al. Efficacy and safety of one anastomosis gastric bypass versus Roux-en-Y gastric bypass at 5 years (YOMEGA): a prospective, open-label, non-inferiority, randomised extension study. Lancet Diabetes Endocrinol. 2024;12:267–76. https://doi.org/10.1016/S2213-8587(24)00035-4.

44. Delko T, Kraljević M, Lazaridis II, Köstler T, Jomard A, Taheri A, et al. Laparoscopic Roux-Y-gastric bypass versus laparoscopic one-anastomosis gastric bypass for obesity: clinical & metabolic results of a prospective randomized controlled trial. Surg Endosc. 2024;38:3875–86. https://doi.org/10.1007/s00464-024-10907-7.

45. Jain M, Tantia O, Goyal G, Chaudhuri T, Khanna S, Majumdar K, et al. LSG vs OAGB: 7-Year Follow-up Data of a Randomised Control Trial and Comparative Outcome Based on BAROS Score. Obes Surg. 2024;34:1295–305. https://doi.org/10.1007/s11695-024-07114-7.

46. Salman MA, Abelsalam A, Nashed GA, Yacoub M, Abdalla A. Long Biliopancreatic Limb Roux-En-Y Gastric Bypass Versus One-Anastomosis Gastric Bypass: a Randomized Controlled Study. Obes Surg. 2023;33:1966–73. https://doi.org/10.1007/s11695-023-06631-1.

47. van Rijswijk A, van Olst N, Meijnikman AS, Acherman YIZ, Bruin SC, van de Laar AW, et al. The effects of laparoscopic Roux-en-Y gastric bypass and one-anastomosis gastric bypass on glycemic control and remission of type 2 diabetes mellitus: study protocol for a multi-center randomized controlled trial (the DIABAR-trial). Trials. 2022;23:900. https://doi.org/10.1186/s13063-022-06762-3.

48. Eskandaros MS, Abbass A, Zaid MH, Darwish AA. Laparoscopic One Anastomosis Gastric Bypass Versus Laparoscopic Roux-en-Y Gastric Bypass Effects on Pre-existing Mild-to-Moderate Gastroesophageal Reflux Disease in Patients with Obesity: a Randomized Controlled Study. Obes Surg. 2021;31:4673–81. https://doi.org/10.1007/s11695-021-05667-5.

49. Level L, Rojas A, Piñango S, Avariano Y. One anastomosis gastric bypass vs. Roux-en-Y gastric bypass: a 5-year follow-up prospective randomized trial. Langenbecks Arch Surg. 2021;406:171–9. https://doi.org/10.1007/s00423-020-01949-1.

50. Robert M, Espalieu P, Pelascini E, Caiazzo R, Sterkers A, Khamphommala L, et al. Efficacy and safety of one anastomosis gastric bypass versus Roux-en-Y gastric bypass for obesity (YOMEGA): a multicentre, randomised, open-label, non-inferiority trial. Lancet. 2019;393:1299–309. https://doi.org/10.1016/S0140-6736(19)30475-1.

51. Saarinen T, Pietiläinen KH, Loimaala A, Ihalainen T, Sammalkorpi H, Penttilä A, et al. Bile Reflux is a Common Finding in the Gastric Pouch After One Anastomosis Gastric Bypass. Obes Surg. 2020;30:875–81. https://doi.org/10.1007/s11695-019-04353-x.

52. Gorini S, Camajani E, Franchi A, Cava E, Gentileschi P, Bellia A, et al. Enhancing nutritional health and patient satisfaction five years after metabolic bariatric surgery with targeted supplementation. J Transl Med. 2025;23:216. https://doi.org/10.1186/s12967-025-06224-9.

53. Sadeghi S, Hosseinpanah F, Khalaj A, Mahdavi M, Valizadeh M, Taheri H, et al. The journey of MASLD: Tracking resolution, relapse, and predictive factors after sleeve gastrectomy and one-anastomosis gastric bypass, a propensity score-matched cohort study. Diabetes Res Clin Pract. 2025;219:111969. https://doi.org/10.1016/j.diabres.2024.111969.

54. Makkapati S, Harsha MS, Palaniappan R. Long-Term Outcome of One-Anastomosis Gastric Bypass: 10-Year Follow-Up of a Single Institution Series. Obes Surg. 2025;35:216–23. https://doi.org/10.1007/s11695-024-07624-4.

55. Pan H-M, Lee W-J, Ser K-H, Soong T-C, Lee M-H, Lin C-H, et al. Impact of metabolic bariatric surgery on outcomes and the 10-year risk of major adverse cardiovascular events during a 7-year period: a retrospective cohort study. Int J Surg. 2024;110:5563–73. https://doi.org/10.1097/JS9.0000000000001631.

56. Sadeghi S, Hosseinpanah F, Khalaj A, Ebadinejad A, Mahdavi M, Valizadeh M, et al. Remission and relapse of diabetes after sleeve gastrectomy and one-anastomosis gastric bypass: The Tehran Obesity Treatment Study. Diabetes Obes Metab. 2024;26:6007–15. https://doi.org/10.1111/dom.15974.

57. Borjas G, Sánchez N, Urdaneta A, Maldonado A, Ramos E, Ferrigni C, et al. Magnetic device in reduced port and single port bariatric surgery: First 170 cases experience. Cir Esp (Engl Ed). 2022;100:614–21. https://doi.org/10.1016/j.cireng.2022.08.016.

58. Bourbour F, Kabir A, Pazouki A, Mokhber S, Kord-Varkaneh H, Găman M-A, et al. Trends in Serum Vitamin D Levels within 12 Months after One Anastomosis Gastric Bypass (OAGB). Obes Surg. 2021;31:3956–65. https://doi.org/10.1007/s11695-021-05434-6.

59. Slagter N, Hopman J, Altenburg AG, de Heide LJM, Jutte EH, Kaijser MA, et al. Applying an Anti-reflux Suture in the One Anastomosis Gastric Bypass to Prevent Biliary Reflux: a Long-Term Observational Study. Obes Surg. 2021;31:2144–52. https://doi.org/10.1007/s11695-021-05238-8.

60. Jimenez J-M, Ruiz-Tovar J, López M, Marc-Hernandez A, Carbajo M-A, Cao M-J, et al. Assessment of body composition in obese patients undergoing one anastomosis gastric bypass: cross-sectional study. Sci Rep. 2020;10:18884. https://doi.org/10.1038/s41598-020-75589-2.

61. Marc-Hernández A, Ruiz-Tovar J, Jimenez JM, Carbajo MA, Lopez M, Cao MJ, et al. Short-Term Changes on Body Composition and Bone Mass After One-Anastomosis Gastric Bypass: a Prospective Observational Study. Obes Surg. 2020;30:3514–21. https://doi.org/10.1007/s11695-020-04603-3.

62. Ruiz-Tovar J, Carbajo MA, Jimenez JM, Luque-de-Leon E, Ortiz-de-Solorzano J, Castro MJ. Are There Ideal Small Bowel Limb Lengths for One-Anastomosis Gastric Bypass (OAGB) to Obtain Optimal Weight Loss and Remission of Comorbidities with Minimal Nutritional Deficiencies? World J Surg. 2020;44:855–62. https://doi.org/10.1007/s00268-019-05243-0.

63. Navarrete Aulestia S, Leyba JL, Navarrete Llopis S, Pulgar V. One Anastomosis Gastric Bypass/Minigastric Bypass in Patients with BMI < 35 kg/m2 and Type 2 Diabetes Mellitus: Preliminary Report. Obes Surg. 2019;29:3987–91. https://doi.org/10.1007/s11695-019-04071-4.

64. Chiappetta S, Schaack HM, Wölnerhannsen B, Stier C, Squillante S, Weiner RA. The Impact of Obesity and Metabolic Surgery on Chronic Inflammation. Obes Surg. 2018;28:3028–40. https://doi.org/10.1007/s11695-018-3320-y.

65. Carbajo MA, Luque-de-León E, Jiménez JM, Ortiz-de-Solórzano J, Pérez-Miranda M, Castro-Alija MJ. Laparoscopic One-Anastomosis Gastric Bypass: Technique, Results, and Long-Term Follow-Up in 1200 Patients. Obes Surg. 2017;27:1153–67. https://doi.org/10.1007/s11695-016-2428-1.

66. Carbajo M, García-Caballero M, Toledano M, Osorio D, García-Lanza C, Carmona JA. One-anastomosis gastric bypass by laparoscopy: results of the first 209 patients. Obes Surg. 2005;15:398–404. https://doi.org/10.1381/0960892053576677.

67. Rutledge R. The mini-gastric bypass: experience with the first 1,274 cases. Obes Surg. 2001;11:276–80. https://doi.org/10.1381/096089201321336584.
